# Non-thermal plasma-catalytic CO_2_ hydrogenation to methanol: mechanisms and catalyst design

**DOI:** 10.1039/d6se00855k

**Published:** 2026-11-03

**Authors:** Maryam Khatibi, Jie Gu, Paul T. Williams, Amin Osatiashtiani

**Affiliations:** a School of Chemical and Process Engineering, University of Leeds Leeds LS2 9JT UK a.osatiashtiani@leeds.ac.uk

## Abstract

The plasma-catalytic hydrogenation of CO_2_ to methanol has attracted growing attention as a potential route for sustainable carbon utilisation and electrified chemical production. Compared with conventional thermocatalytic processes, which are constrained by unfavourable thermodynamic–kinetic trade-offs, non-thermal plasma enables CO_2_ activation under significantly milder conditions through the generation of energetic electrons, radicals, ions, and vibrationally excited species. When integrated with heterogeneous catalysts, these plasma environments can activate CO_2_ and H_2_ under mild conditions and improve methanol productivity. Catalyst composition and plasma operating conditions play a decisive role in directing selectivity toward methanol over competing products. This review critically discusses the current state of non-thermal plasma-assisted CO_2_ hydrogenation to methanol, with emphasis on plasma fundamentals, mechanistic understanding, catalyst design, and the influence of key operating parameters. Based on surveying the research landscape, we discuss the remaining scientific and technological challenges and highlight key opportunities for advancing plasma-catalytic methanol synthesis toward practical implementation.

## Abstract 2

This review provides a comprehensive overview of recent advances in non-thermal plasma-catalytic CO_2_ hydrogenation to methanol, covering plasma fundamentals, reaction mechanisms, catalyst design, operating parameters, challenges and future outlook.

## Graphical abstract

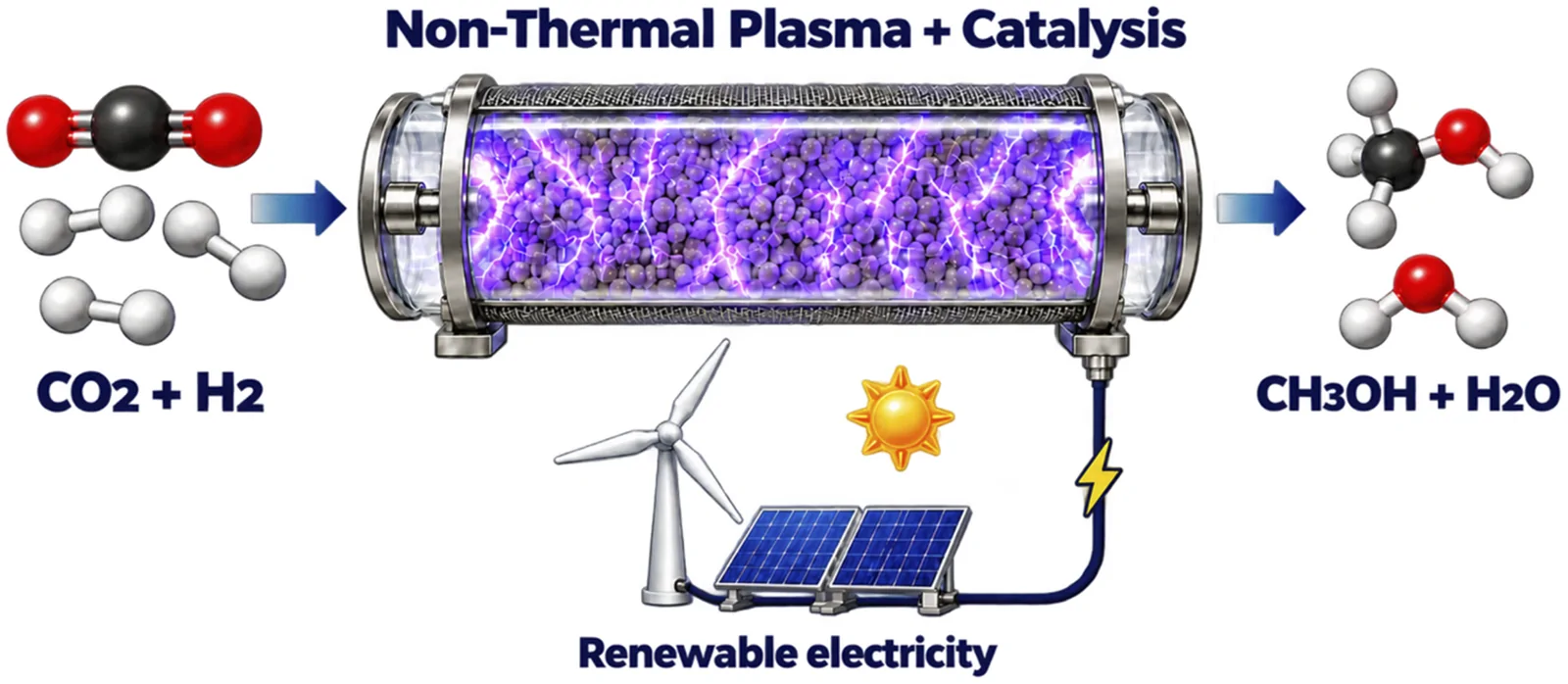

## 1. Introduction

Carbon dioxide is an abundant carbon resource and one of the most stable molecules in nature. This combination makes CO_2_ both an attractive C_1_ feedstock and a demanding target for chemical transformation. As an inexpensive, widely available, and non-toxic carbon source, CO_2_ offers clear potential for the sustainable production of fuels and value-added chemicals.^1,2^ However, its high thermodynamic stability and kinetic inertness present major barriers to activation, often requiring significant energy input or highly effective catalytic systems. The efficient utilisation of CO_2_ therefore depends on the development of innovative activation strategies capable of overcoming these intrinsic limitations.

Among the possible products derived from CO_2_, methanol has attracted particular attention because of its versatility and established industrial importance. Methanol is a major commodity chemical, with global production reaching 111 million tonnes in 2022 and continued growth projected.^3^ It is widely used in the manufacture of formaldehyde, acetic acid, olefins, and a range of solvents and specialty chemicals.^4^ Apart from its role as a chemical feedstock, methanol is also considered a promising fuel due to its high octane rating and its potential use as a gasoline additive in internal combustion engines.^5,6^ Methanol can also act as a liquid hydrogen carrier, enabling easier storage and transportation, and can serve as an intermediate for the production of synthetic hydrocarbons and sustainable fuels.^7^ As a fuel, its combustion can generate lower emissions of pollutants such as NO_*x*_ and SO_*x*_ compared with many conventional fuels.^8,9^ These advantages make methanol synthesis from CO_2_ an attractive route for combining carbon utilisation with renewable energy storage and sustainable chemical production.

Despite its strong potential, the catalytic hydrogenation of CO_2_ to methanol remains challenging under conventional thermocatalytic conditions. Methanol synthesis is exothermic and thermodynamically favoured at lower temperatures and elevated pressures, yet the inertness of CO_2_ often requires higher temperatures to achieve practical reaction rates. This creates an inherent trade-off, as increasing temperature can suppress methanol equilibrium yield and promote competing pathways such as the reverse water-gas shift (RWGS) reaction.^10^ As a result, reported thermocatalytic CO_2_-to-methanol systems commonly employ carefully optimised catalyst formulations and elevated pressure/temperature conditions to balance activity, selectivity, and productivity.^11,12^ In addition, the formation of water during reaction can influence catalyst performance, surface chemistry, and long-term stability.^13,14^

These challenges are accompanied by wider efforts to transition the chemical industry towards process electrification. This transition is driven by the need to reduce carbon emissions from energy-intensive manufacturing and to make better use of the increasing supply of renewable electricity.^15^ Conventional thermocatalytic processes rely on heat generated by burning fossil fuels, which contributes significantly to industrial CO_2_ emissions and energy losses. Electrified technologies, such as resistive heating, inductive heating, microwave-assisted catalysis, and plasma–driven processes, provide an alternative by using electrical energy directly instead of fossil fuel combustion.^16^ As electricity from wind, solar, and other renewable sources becomes more widely available, these technologies offer a more sustainable approach to chemical production. This transition is especially important for reactions such as CO_2_ hydrogenation, where conventional thermal processes often require a compromise between activity and selectivity, as discussed above.

In this context, plasma has emerged as a promising alternative strategy for CO_2_ activation. Plasma is often described as the fourth state of matter and consists of a partially ionised gas containing free electrons, ions, neutral molecules, radicals, and excited species.^17^ Plasma can be broadly classified into thermal and non-thermal systems. Thermal plasma is characterised by near-equilibrium temperatures, meaning that electrons, ions, and neutral species are at similar temperatures, resulting in a uniformly hot gas. In contrast, non-thermal plasma (NTP) contains highly energetic electrons in the range of 1–10 eV, corresponding to effective temperatures of 10^4^–10^5^ K, whereas the bulk gas remains near ambient temperature or only moderately heated.^18,19^ These energetic electrons can excite, ionise, or dissociate reactants such as CO_2_ and H_2_ through collision processes without heating the entire reactor volume. As a result, reactive species such as radicals, ions, metastables, and vibrationally excited molecules are generated and may participate in subsequent gas-phase and surface reactions.^20^ This makes NTP especially attractive for catalytic applications. When combined with heterogeneous catalysts, NTP can also modify catalyst surfaces through local electric fields, surface charging, defect generation, changes in oxidation state, enhanced adsorption, or the creation of additional active sites.^21^ This creates a unique reaction environment in which plasma-phase activation and catalytic surface chemistry operate cooperatively. As a result, kinetic barriers can be reduced and stable molecules such as CO_2_ and H_2_ can be activated under milder conditions.

Despite increasing interest in plasma-assisted CO_2_ hydrogenation, the selective formation of methanol remains less developed than competing routes that favour carbon monoxide or methane. Important questions remain regarding reaction pathways, catalyst composition, plasma reactor configuration, the role of operating variables in determining performance, and energy efficiency. An earlier review provided an overview of the initial developments in this field, covering studies published mainly up to 2021, and readers are referred to that work for further background.^22^ Since then, significant progress has been made in catalyst development and understanding plasma-catalyst interactions. This review focuses on advances reported between 2022 and 2026, with particular emphasis on methanol formation pathways, plasma-specific effects, catalyst design and operating parameters. It also critically examines energy efficiency, techno-economic feasibility and challenges associated with process scale-up, highlighting remaining research gaps and opportunities for future development.

## 2. Fundamentals of non-thermal plasma for CO_2_ hydrogenation

The performance of NTP-assisted CO_2_ hydrogenation systems is strongly influenced by the characteristics of the plasma discharge, reactor configuration, reactant activation pathways, and plasma-catalyst interactions.^23^ Unlike conventional thermocatalytic processes, NTP systems involve the simultaneous interaction of plasma physics, gas-phase chemistry, and heterogeneous surface reactions. These interactions determine the formation and distribution of reactive species, energy transfer behaviour, catalyst surface properties, and the activity and selectivity of the reaction system.^24,25^ Therefore, understanding the fundamental aspects of plasma generation, molecular activation, and plasma-catalyst synergy is essential for the rational design and optimisation of plasma-assisted methanol synthesis processes.

Several types of NTP reactors have been investigated for plasma-assisted CO_2_ conversion, including dielectric barrier discharge (DBD),^26^ corona discharge,^27,28^ glow discharge,^29,30^ microwave plasma,^31^ and gliding arc systems.^32^ These plasma sources differ in discharge behaviour, electron density, gas temperature, electric field distribution, and energy transfer characteristics, all of which influence reactant activation and product selectivity. Among these, DBD reactors are the most widely studied for plasma-catalytic CO_2_ hydrogenation to methanol due to their relatively simple design, atmospheric-pressure operation, low gas temperatures, and compatibility with catalyst integration.

In a typical DBD reactor, two electrodes are separated by a small gap through which the reactant gases flow, as shown in Fig. 1. At least one of the electrodes is covered by an insulating dielectric material, such as quartz, glass, ceramic, or alumina. When a high alternating voltage is applied between the electrodes, the electric field in the gas gap becomes strong enough to ionise the gas molecules and generate plasma. Rather than forming a single continuous spark, the dielectric layer limits the electrical current and causes the plasma to appear as numerous short-lived and spatially distributed microdischarges throughout the reactor volume. This prevents the transition to hot arc discharges and helps maintain a stable NTP regime.^19^ As a result, DBD reactors can operate at atmospheric pressure and generate highly energetic electrons that activate CO_2_ and H_2_ molecules without significantly increasing the bulk gas temperature.

**Fig. 1:**
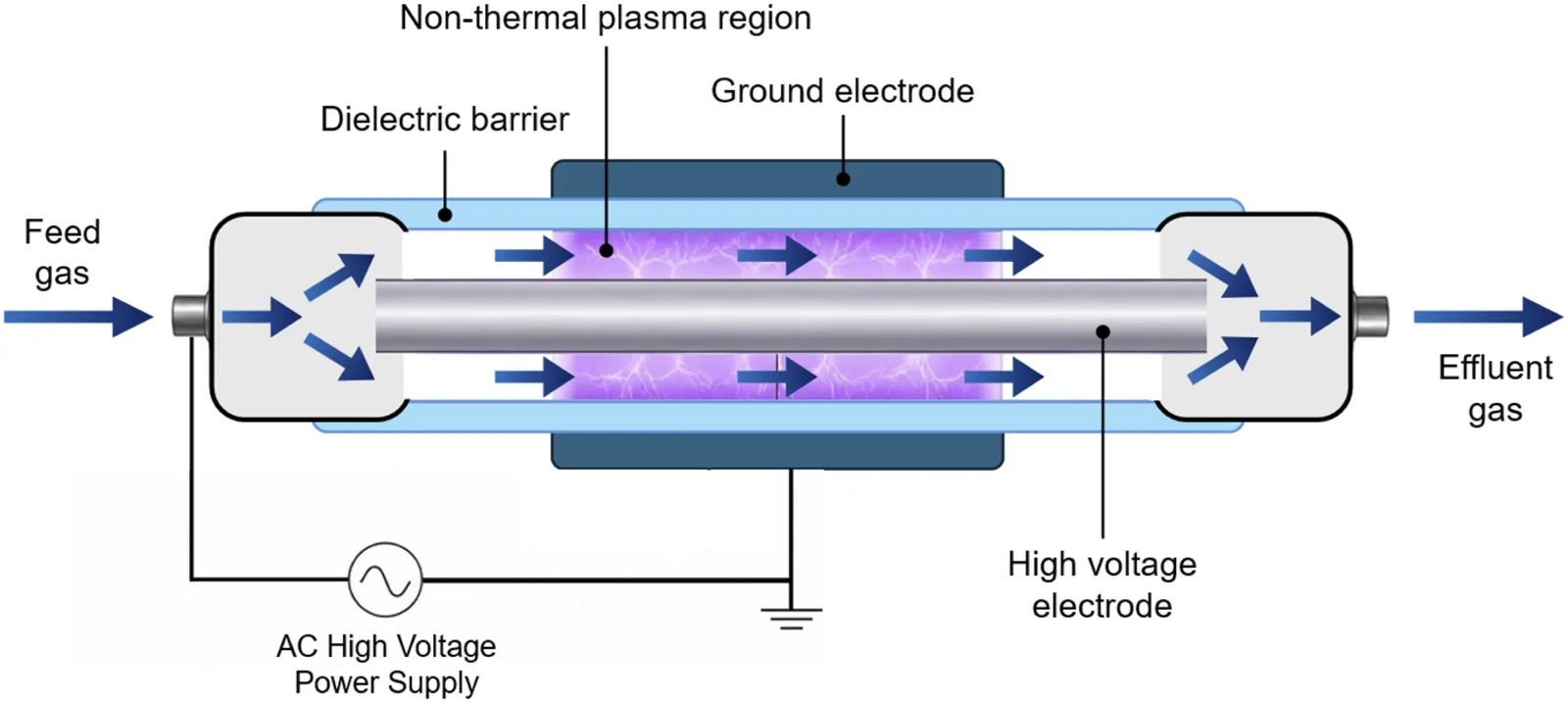
Schematic illustration of a coaxial dielectric barrier discharge (DBD) plasma reactor. Reproduced from ref. 33 under the terms of the Creative Commons CC BY licence.

Different reactor configurations have been employed in plasma-catalytic CO_2_ hydrogenation studies. From the perspective of catalyst placement, these systems are commonly classified as plasma-alone, post-plasma catalysis (PPC, also referred to as a two-stage configuration), and in-plasma catalysis (IPC, also known as a one-stage configuration), as shown in Fig. 2.^23,34,35^ In plasma-alone systems, reactions occur solely through plasma-generated species without the presence of a catalyst. In post-plasma catalysis, the catalyst is positioned downstream of the discharge region, so only sufficiently long-lived plasma-generated species can interact with the catalyst surface, which limits the potential for plasma-catalyst synergistic effects.^34,35^ In contrast, in-plasma catalysis, places the catalyst directly within the discharge zone, allowing stronger interaction between the plasma and catalyst surface.^34,35^ The presence of the catalyst can significantly influence plasma behaviour by modifying local electric fields, altering discharge propagation, and affecting microdischarge formation. At the same time, plasma exposure can influence catalyst surface properties, as mentioned above. These mutual plasma-catalyst interactions may contribute to enhanced reactant activation and altered reaction pathways compared with conventional thermal catalysis.^23,34,35^

**Fig. 2:**
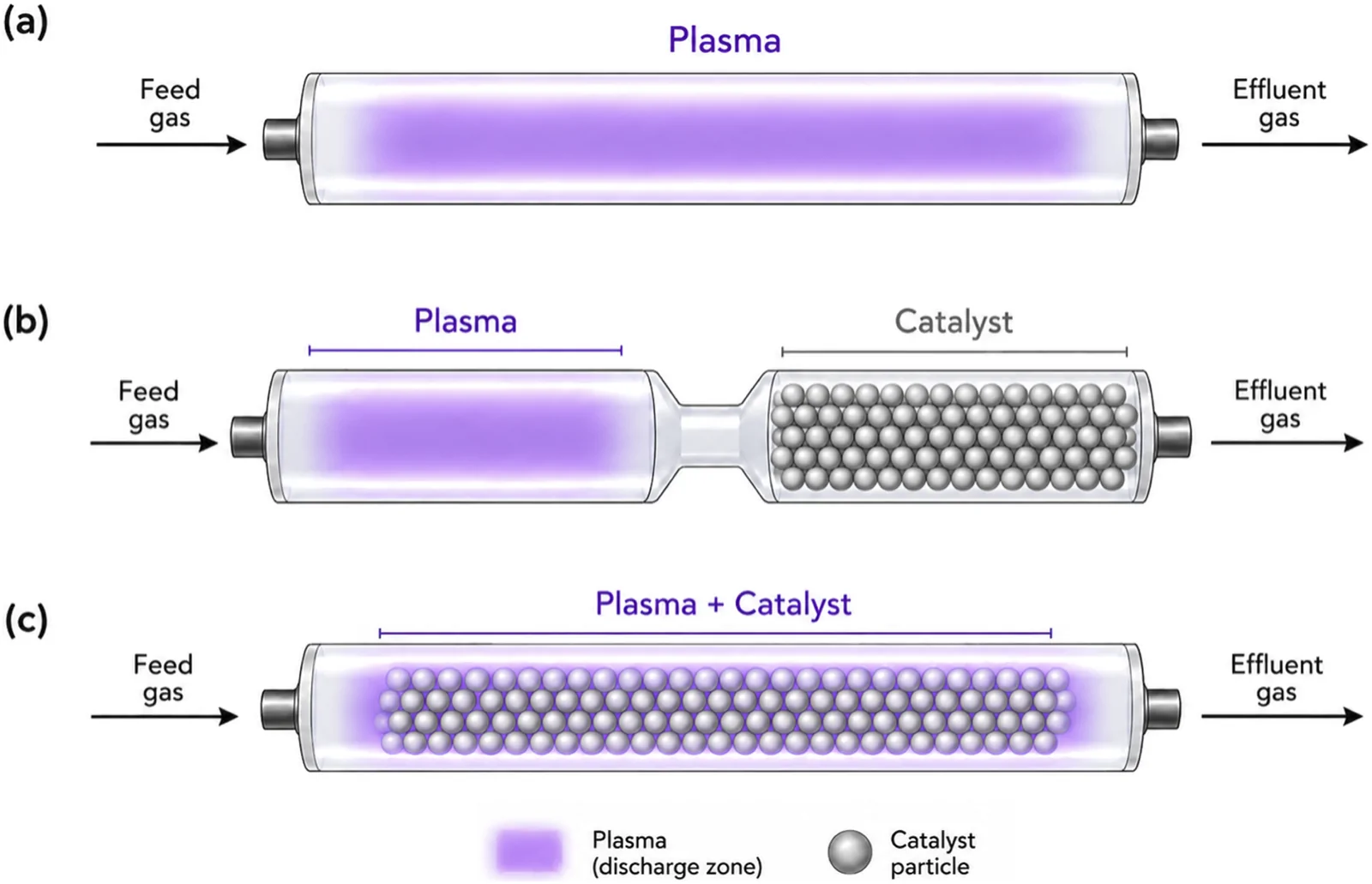
Schematic illustration of different plasma reactor configurations used in plasma-catalysis: (a) plasma-only reactor, (b) post-plasma catalysis (PPC), and (c) in-plasma catalysis (IPC) reactor with catalyst packed within the plasma discharge zone. The dielectric tube, external electrodes, plasma discharge region, and catalyst particles are indicated schematically.

Within in-plasma catalysis DBD systems, catalysts can be introduced using several structural approaches: packed-bed configurations, in which catalyst or dielectric particles fill the discharge gap directly; coated configurations, where catalytic material is deposited onto the dielectric barrier, reactor wall, or electrode surface; honeycomb or monolith-supported catalysts; and foam-based catalytic structures.^19,36^ These integration strategies differ substantially in plasma-catalyst contact area, pressure drop, mass transfer characteristics, and electric field distribution, and can consequently have a strong influence on catalytic performance and product selectivity. Among these, packed-bed DBD reactors are the most widely studied configuration in plasma catalysis. In these reactors, catalyst particles or dielectric packing materials are placed directly within the discharge gap. This arrangement enables close plasma-catalyst contact and enhances local electric fields at particle contact points and therefore, microdischarges can form more readily.^19,34^ These microdischarges in turn promote the generation of reactive species, excited states, and energetic electrons in the immediate vicinity of the catalyst surface, and it is through these non-equilibrium interactions that plasma exerts its most significant influence on reaction mechanisms and product formation pathways.

## 3. Reaction mechanisms and methanol formation pathways

Understanding the reaction mechanisms involved in plasma-catalytic CO_2_ hydrogenation to methanol is essential for interpreting the plasma-catalyst performance, product selectivity as well as guiding catalyst design. Compared with conventional thermocatalytic systems, NTP introduces additional reactive species, excited states, and non-equilibrium effects that can alter activation pathways and intermediate formation. Consequently, several mechanistic pathways have been proposed for methanol synthesis under plasma conditions. The following sections discuss the main reaction pathways and intermediates reported in the literature.

### 3.1. Non-thermal plasma without catalyst

Wang *et al.* proposed a reaction mechanism for NTP CO_2_ hydrogenation to methanol without a catalyst in a DBD reactor.^26^ They proposed that the mechanism proceeds through gas-phase reactions initiated by electron impact. Energetic electrons primarily dissociate CO_2_ into CO and O radicals. Additional CO formation may also occur through reactions involving vibrationally excited CO_2_ with O or H radicals, although this pathway was considered less significant. Once CO is produced, it undergoes stepwise hydrogenation. CO reacts with H to form HCO, which recombines to produce H_2_CO (formaldehyde), followed by successive hydrogenation to H_3_CO and finally CH_3_OH. However, they identified that the formation of H_2_CO is the critical limiting step because HCO can also undergo a competitive reaction with H to regenerate CO and H_2_, resulting in a reduced methanol selectivity.

Complementary modelling studies have provided further insight into plasma-phase reaction kinetics and reactive species evolution. Chen *et al.* used a zero-dimensional plasma kinetic model to investigate CO_2_ hydrogenation under NTP conditions.^37^ Their simulations showed that increasing the H_2_ : CO_2_ ratio increased electron density and electron temperature, promoting the formation of reactive radicals involved in hydrogenation reactions. Reaction-flow analysis identified interconnected pathways involving CO, CH_2_O, CH_3_OH, CH_4_, and H_2_O, indicating that methanol synthesis proceeds through a complex radical-mediated reaction network rather than a single dominant elementary step. The study further proposed that methanol formation mainly proceeds through recombination of CH_3_ and OH radicals, followed by hydrogenation of CH_2_OH intermediates.

Liao *et al.* further analysed atmospheric-pressure DBD plasma using a two-dimensional fluid model and identified H, O, and CO as the dominant reactive plasma species generated during CO_2_ hydrogenation.^38^ Their simulations showed that H radicals are formed mainly through electron-impact dissociation of H_2_, whereas O and CO originate primarily from electron-induced CO_2_ dissociation. The CO_2_ feed ratio also affected the discharge characteristics. Increasing the CO_2_ fraction led to greater space-charge accumulation and a stronger axial electric field. This increased the streamer propagation velocity (1.45 × 10^6^ compared with 0.7 × 10^6^ m s^−1^) and discharge current. A higher H_2_ fraction promoted the formation of hydrogen-related species, including H_2_^+^ ions, but reduced the concentration of CO_2_-derived species.

Table 1 summarises the performance of non-catalytic DBD plasma reactors for CO_2_ hydrogenation to methanol. In general, non-catalytic NTP systems alone are insufficient for achieving practically relevant methanol yields. The incorporation of heterogeneous catalysts is therefore necessary to enhance CO_2_ conversion and direct the reaction towards methanol. The following section discusses the role of heterogeneous catalysts in plasma-assisted CO_2_ hydrogenation. The main non-catalytic NTP CO_2_-to-methanol pathways are summarised in Fig. 3.

**Table 1:** Performance of non-catalytic DBD plasma reactor for CO_2_-to-methanol conversion

*X*_CO_2__ a (%)	S_MeOH_^b^ (%)	Y_MeOH_^c^ (%)	Reaction condition	Ref.
13.2	54	7.2	CO_2_ : H_2_ = 1 : 3	26
			Total flow rate = 40 mL min^−1^	
			Reaction temperature = 30 °C	
			Frequency = 9 kHz	
			Discharge power = 10 W	
6.1	3.7	0.23	CO_2_ : H_2_ = 1 : 3	39
			Total flow rate = 72 mL min^−1^	
			Circulating water ≈ 60 °C	
			Frequency = 9.5 kHz	
			Input power = 26 W	
5	0.1	∼0	CO_2_ : H_2_ = 1 : 3	40
			Total flow rate = 40 mL min^−1^	
			Ambient temperature	
			Discharge power = 5 W	
3.3	7.5	0.25	CO_2_ : H_2_ = 1 : 3	41
			Total flow rate: 72 mL min^−1^	
			Circulating water = 30 °C	
			Frequency: 9.2 kHz	
			Input power: 24 W	
∼0.5	3.7^d^	∼0.02	Feed: 20 vol% CO_2_, 60 vol% H_2_,20 vol% Ar	42
			Total flow rate: 50 mL min^−1^	
			Reaction temperature = 29 °C	
			Applied peak voltage: 10.2 kV	
			Frequency: 7.7 kHz	
			Plasma power: 11 W	

a CO_2_ conversion.

b Methanol selectivity.

c Methanol yield.

d CO-free selectivity.

**Fig. 3:**
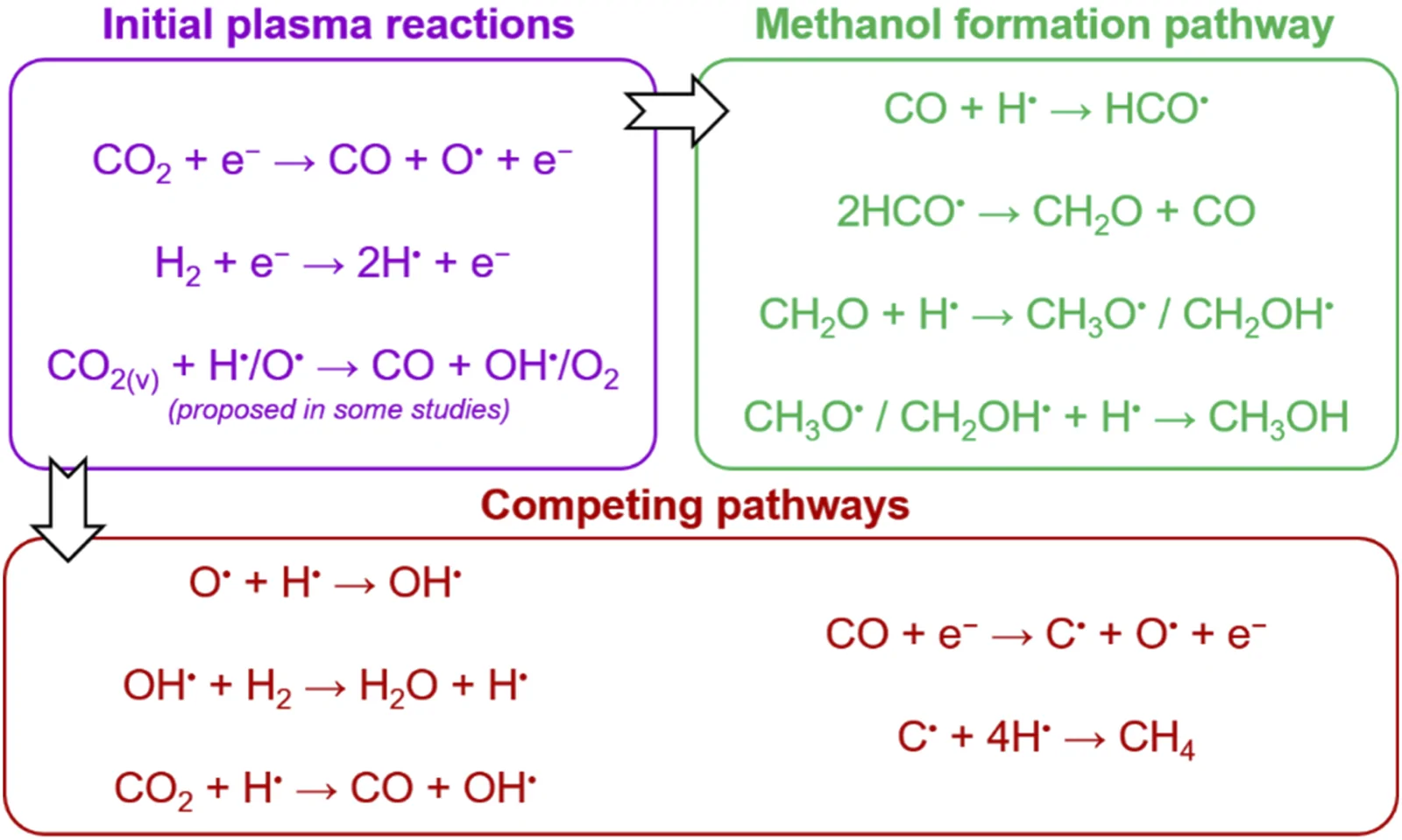
The proposed main reaction pathways for methanol production in non-thermal plasma CO_2_ hydrogenation without a catalyst, based on ref. 26 and 43.

### 3.2. Non-thermal plasma with catalyst

Introducing a heterogeneous catalyst into the discharge zone fundamentally changes the reaction environment. The interaction between plasma and catalyst is bidirectional. Plasma can modify catalyst surface properties through electric field enhancement, defect generation, surface charging, and changes in oxidation state. In turn, the catalyst can influence discharge behaviour and microdischarge formation.^44,45^ The introduction of a catalyst also fundamentally changes the dominant reaction mechanism compared with plasma-only systems. Rather than relying exclusively on gas-phase hydrogenation, plasma-catalytic systems combine both gas-phase and surface reactions. The catalyst provides adsorption sites for short-lived plasma-generated intermediates, enabling their activation and subsequent hydrogenation through heterogeneous reaction pathways. As a result, plasma-catalytic systems combine two interconnected activation environments: (i) the gas phase, where plasma generates reactive species, and (ii) the catalyst surface, where these species are adsorbed and converted into products.

Across the literature, four principal pathways have been proposed for the plasma-catalytic CO_2_ hydrogenation to methanol; (1) the formate route, in which CO_2_ is hydrogenated stepwise through formate and methoxy intermediates; (2) the reverse water-gas shift (RWGS) followed by CO hydrogenation route, in which CO_2_ undergoes RWGS to form CO and its subsequent surface hydrogenation to methanol; (3) the carboxyl route, which proceeds through a surface carboxyl intermediate; and (4) the carbide route, in which adsorbed CO loses oxygen to form a surface carbon species that is progressively hydrogenated. In most plasma-catalytic systems, these pathways are not mutually exclusive and operate concurrently, with their relative contributions governed by catalyst composition, surface basicity, oxygen vacancy density, and plasma discharge parameters.

Many of the adsorbed intermediates proposed in plasma-catalytic CO_2_ hydrogenation are similar to those reported in thermocatalytic systems. Consequently, several downstream surface hydrogenation steps are often interpreted using mechanistic frameworks analogous to conventional heterogeneous catalysis. However, it must be noted that plasma-specific effects, including radical-assisted reactions, non-equilibrium conditions, and dynamic catalyst surface modification, may alter the relative importance, kinetics, and selectivity of these pathways. Fig. 4 summarises the main proposed reaction pathways for plasma-catalytic CO_2_ hydrogenation to methanol. Each of these pathways are discussed in more detail below.

**Fig. 4:**
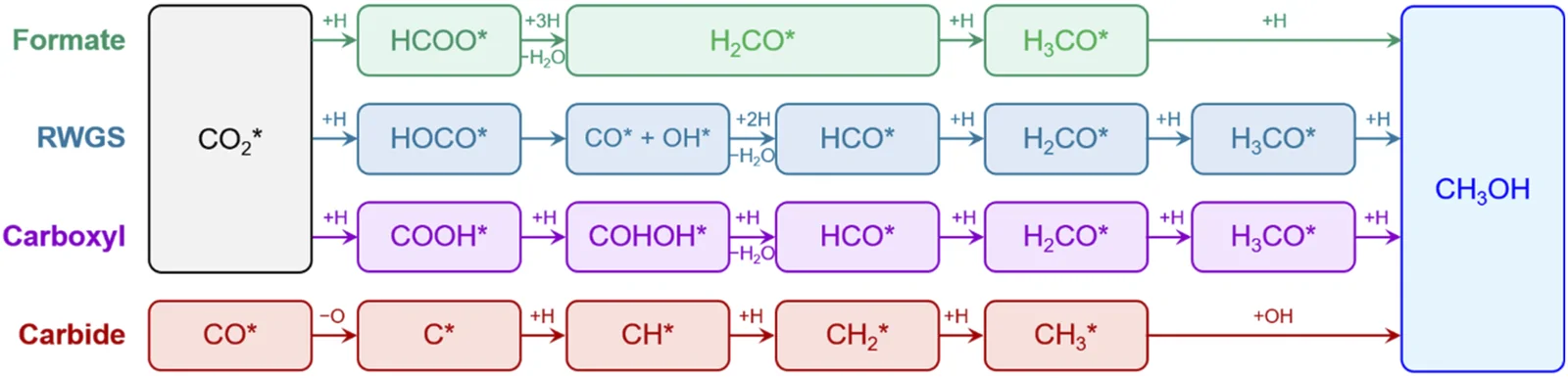
Proposed plasma–catalytic pathways for CO_2_ hydrogenation to methanol, including the formate route, RWGS-mediated CO hydrogenation route, carboxyl route, and carbide route, based on ref. 41 and 46–50. Some arrows represent multiple elementary steps.

#### 3.2.1 Formate route (HCOO*)

The formate route is the most widely reported pathway in plasma-catalytic methanol synthesis and has been identified across a broad range of catalyst compositions. In this mechanism, plasma-generated reactive species activate CO_2_, leading to the formation of adsorbed formate (HCOO*), which undergoes further stepwise hydrogenation to methanol through formaldehyde- and methoxy-type intermediates. A notable difference from thermal catalysis was observed by Zhang *et al.* using *in situ* DRIFTS and gas-phase FTIR spectroscopy over MnO_*x*_/ZrO_2_ catalysts.^40^ Under thermal catalytic conditions, the reaction was dominated by the progressive accumulation of (bi)carbonate species. In contrast, under DBD plasma conditions, the intensities of these species did not increase substantially with time, and HCOO* and CH_3_O* bands appeared rapidly (Fig. 5). They suggested that plasma conditions altered the surface intermediate distribution and promoted the formation of intermediates associated with methanol synthesis. Plasma-generated species such as 
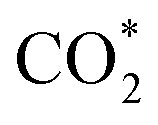
, CO_2_^+^, and H radicals were proposed to facilitate HCOO* formation, and MnO_*x*_ was suggested to promote the further hydrogenation of HCOO* toward CH_3_O*, a step that was not effectively observed over ZrO_2_ alone.

**Fig. 5:**
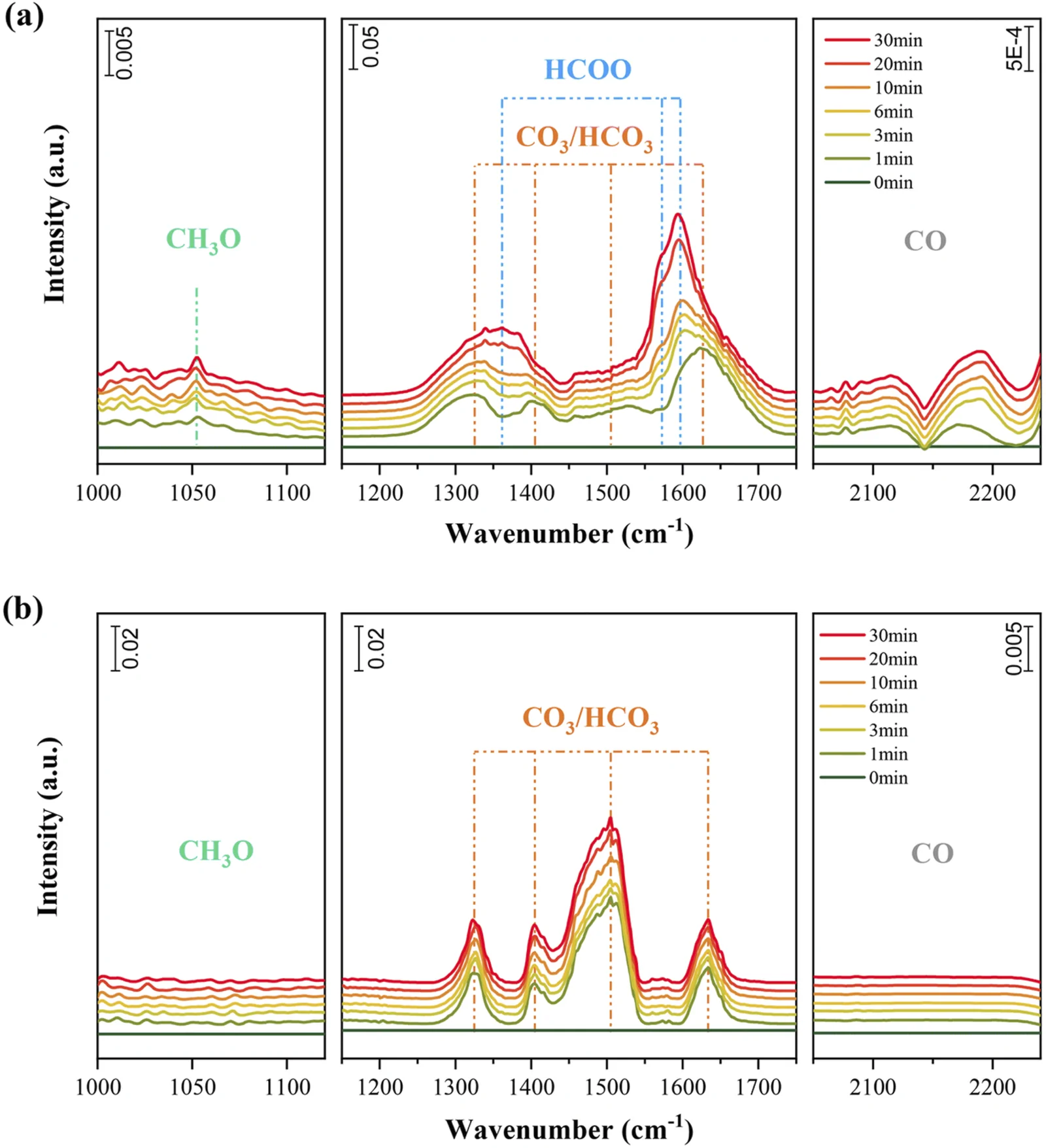
*In situ* DRIFT spectra of (a) plasma-catalytic and (b) thermocatalytic CO_2_ hydrogenation over MnO_*x*_/ZrO_2_. Reproduced from ref. 40, with permission from the Royal Society of Chemistry, copyright 2023.

This formate-mediated mechanism has also been identified over noble and base metal catalysts. Wang *et al.* proposed multiple catalyst–surface pathways over Cu/Al_2_O_3_ and Pt/Al_2_O_3_, including the formate route, and an RWGS-derived CO hydrogenation pathway, as shown in Fig. 6.^26^ Here, plasma-generated reactive species, such as vibrationally excited CO_2_(v), CO, H and O radicals, were proposed to initiate both gas-phase and surface reactions. It was suggested that CO_2_(v) may adsorb more favourably than ground-state CO_2_, lowering the barrier for HCOO* and HOCO* formation, although the authors also noted that excited CO_2_ species may be quenched or relaxed at the surface and that this contribution could be minor.

**Fig. 6:**
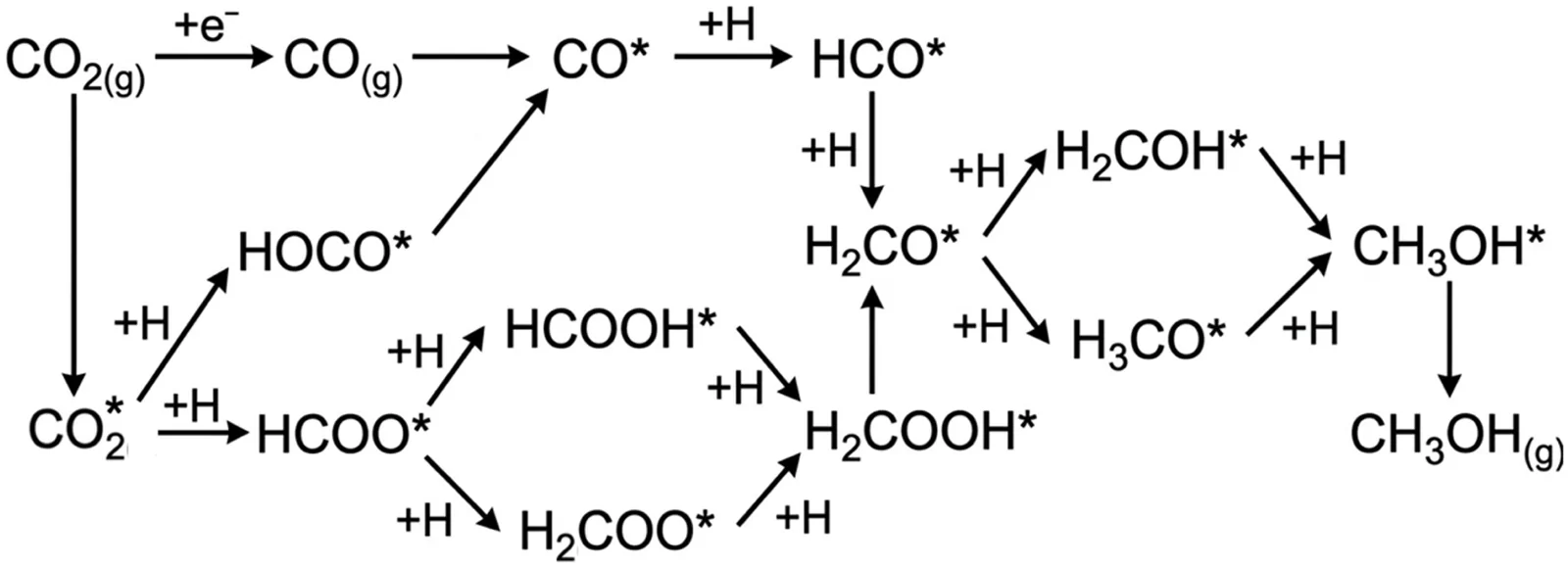
Proposed reaction pathways by Wang *et al.* for plasma-catalytic CO_2_ hydrogenation to methanol. Adapted with permission from ref. 26. Copyright 2018 American Chemical Society.

Consistent with these findings, Chen *et al.* reported a predominantly formate mechanism over CuO–MgO/Beta zeolite.^41^ They suggested that MgO promoted CO_2_ adsorption as carbonate species. Upon introduction of CuO, the dominant surface intermediates shifted from carbonates to formate (HCOO*) and methoxy (CH_3_O*) species, accompanied by increasing methanol-related bands and suppression of gas-phase CO signals. These findings suggested synergistic roles of CuO and MgO in promoting hydrogenation of carbonate-derived intermediates toward methanol formation.

Further evidence for the formate pathway was provided by Chen *et al.*, who examined plasma-catalytic hydrogenation of CO_2_ over Cu–Ni/γ-Al_2_O_3_–CeO_2_.^51^ Using *in situ* FTIR, bicarbonate, formate, methoxy, and adsorbed CO species were detected. Bicarbonates formed rapidly at the early stages and were subsequently converted into formates, followed by hydrogenation into methoxy and methanol species. The progressive increase in formate intensity and the behaviour of methoxies suggested that methanol synthesis mainly proceeded through a formate pathway (
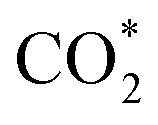
 → HCOO* → HCOOH* → 
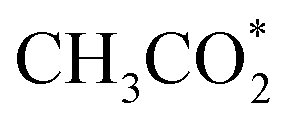
 → CH_2_O* → CH_3_O* → CH_3_OH*). Ni incorporation enhanced H_2_ activation and H spillover, which helped accelerating hydrogenation of formate, methoxy, and carbonyl intermediates and preventing CH_3_O* accumulation. CeO_2_ on the other hand promoted CO_2_ activation and formate formation through oxygen vacancies and weak basic sites.

The formate pathway has also been reported over iron-based catalysts. Meng *et al.* proposed that plasma-catalytic CO_2_ hydrogenation over Fe_2_O_3_/γ-Al_2_O_3_ mainly proceeded through a formate pathway (Fig. 7).^52^ The Fe_2_O_3_/γ-Al_2_O_3_ catalyst exhibited significantly higher intensities of HCOO*, CH_3_O*, and 
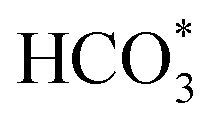
 species than γ-Al_2_O_3_ alone, indicating that Fe sites promoted CO_2_ activation and hydrogenation. They also proposed that chemisorbed oxygen species and Fe(iii)/Fe(ii) redox cycling generated oxygen vacancies that facilitated CO_2_ activation and C=O bond dissociation. At higher plasma powers, linearly adsorbed CO species were detected, suggesting the additional contribution of an RWGS-assisted CO hydrogenation pathway under more energetic discharge conditions.

**Fig. 7:**
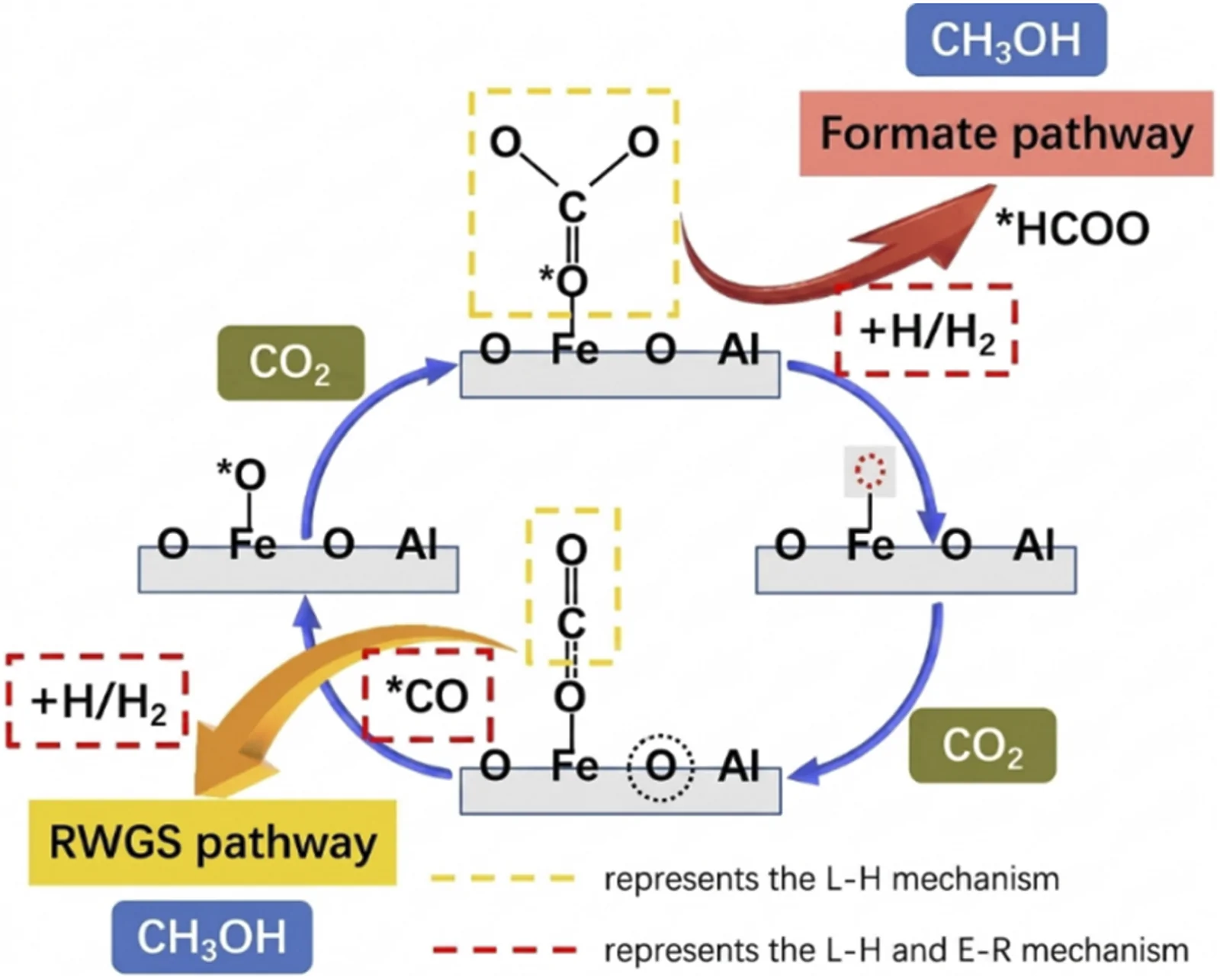
Reaction mechanism of CO_2_ hydrogenation to methanol over Fe_2_O_3_/γ-Al_2_O_3_ catalyst proposed by Meng *et al.* Reproduced from ref. 52 with permission from Wiley, copyright 2023.

Catalyst surface modification can further influence the proposed methanol formation mechanism. Pan *et al.* investigated polytetrafluoroethylene (PTFE)-modified Co_3_O_4_/Al_2_O_3_ and, based on kinetic simulations and *in situ* plasma DRIFTS proposed a dominant formate-mediated pathway.^48^ They proposed that γ-Al_2_O_3_ first promotes CO_2_ adsorption and carbonate formation, then Co_3_O_4_ active sites convert carbonates into formates, followed by further hydrogenation to methoxy and methanol. PTFE incorporation enhanced surface hydrophobicity, accelerated H_2_O desorption, resulting in a reduced competitive adsorption by water species, and enhanced formation and hydrogenation of formate and methoxy intermediates.

The interplay between CO* and formate hydrogenation pathways was further explored by Fang *et al.*, who used *in situ* DRIFTS to investigate plasma-catalytic CO_2_ hydrogenation over Cu, Co_3_O_4_, and Cu–Co catalysts.^42^ They identified adsorbed CO*, formate, and methoxy species as the main surface intermediates. Their results suggested that Cu mainly promoted methanol formation through a CO* hydrogenation pathway, since CO* species were continuously consumed to form CH_3_O*, whereas HCOO* accumulated without effective further hydrogenation. In contrast, Co_3_O_4_ facilitated both CO* and HCOO* hydrogenation pathways, with stronger formate signals indicating a greater contribution from the formate route. The bimetallic Cu–Co catalyst showed the highest CH_3_O* intensity and effective consumption of HCOO*, indicating improved hydrogenation of both CO* and formate intermediates. This was attributed to interfacial electron transfer and hydrogen spillover from Cu to Co_3_O_4_, which increased hydrogen availability on Co_3_O_4_ sites and promoted the hydrogenation of HCOO* and CO* toward methoxy intermediates and ultimately methanol.

In addition to these experimental studies, Michiels *et al.* modelled the quantitative impact of plasma on methanol synthesis kinetics using a microkinetic framework for Cu(111).^50^ Their model predicted a six-to-seven order of magnitude enhancement in methanol turnover frequency relative to thermal catalysis. This was attributed to the higher production through the formate path *via* HCOOH* caused by the increased H* coverage. CO was also proposed to contribute substantially to methanol synthesis by forming HCO* intermediates that subsequently convert into HCOO* species within the formate pathway. CO hydrogenation *via* HCO* and H_2_CO* also contributes to CH_3_OH formation. The model further predicted O* surface poisoning when the H radical partial pressure fell below approximately half the O radical partial pressure. These findings suggest that sufficient H radical availability relative to O, together with CO availability, can promote methanol formation. Although the predicted poisoning threshold is specific to the simulated Cu(111) surface and the assumed plasma-species partial pressures, the results highlight the importance of optimising the CO_2_ feed ratio and plasma operating conditions to favour H radical and CO generation.

The energetic basis for the enhanced formate pathway was further quantified by Cui *et al.* using Density Functional Theory (DFT) calculations on a γ-Al_2_O_3_-supported Cu_13_ cluster.^39^ They proposed that methanol formation over Cu/γ-Al_2_O_3_ proceeds through both the formate and RWGS/CO-hydrogenation pathways. The formate route was more favourable due to lower activation barriers. One of their key findings was that plasma-generated H radicals participating *via* Eley–Rideal (ER) reactions reduced the HCOOH* formation barrier from 1.32 to 0.17 eV and the CH_3_OH formation barrier from 1.25 to 0.15 eV.

#### 3.2.2 RWGS + CO hydrogenation route

The RWGS + CO hydrogenation route is another important methanol formation pathway in plasma-catalytic CO_2_ hydrogenation. The defining feature of this mechanism is that CO acts as the key intermediate between CO_2_ activation and methanol formation. In this route, CO generated through plasma-phase dissociation or surface RWGS is adsorbed onto the catalyst surface and subsequently hydrogenated stepwise to methanol, although CO desorption can also occur as a competing pathway. As noted in the context of the formate pathway, Wang *et al.* proposed such an RWGS-assisted CO hydrogenation pathway over Cu/Al_2_O_3_ and Pt/Al_2_O_3_.^26^ They proposed adsorbed CO_2_ initially undergoes hydrogenation to form HOCO*, which subsequently dissociates into CO* and OH*. The resulting CO* can either desorb or undergo sequential hydrogenation through HCO*, H_2_CO*, and methoxy intermediates toward CH_3_OH formation. Based on previous theoretical studies, the authors suggested that Pt nanoparticles preferentially favour initial HOCO* formation more than the formate route.

Further support for this pathway was provided by Ronda-Lloret *et al.*, who proposed that the RWGS + CO hydrogenation route can be initiated by plasma-assisted CO_2_ dissociation to CO in the gas phase, followed by adsorption of CO on the catalyst surface and subsequent hydrogenation toward methanol.^43^ They also suggested that adsorbed CO_2_ can form HOCO intermediates, whose decomposition to CO initiates the RWGS pathway. Both the RWGS-derived CO route and the formate route were proposed to converge through H_2_CO and methoxy intermediates before methanol formation. Their results further showed that MgO and Al_2_O_3_ mainly promoted CO formation because of their strong CO_2_ adsorption capacity but weak affinity toward CO and hydrogen species. In contrast, CoO_*x*_/MgO catalysts enhanced methanol formation, attributed to increased availability of adsorbed CO and hydrogen species for subsequent methanol synthesis steps.

The contribution of CO hydrogenation to methanol formation was further examined by Mei *et al.* over Cu–ZnO/Al_2_O_3_ (CZA) foam ceramics.^53^ They suggested that plasma first promotes CO formation through electron-impact dissociation of CO_2_. The generated CO can then adsorb on the catalyst surface and undergo sequential hydrogenation through CHO*, CH_2_O*, and CH_3_O* intermediates to produce methanol (Fig. 8). They also proposed that adsorbed HOCO* intermediates may dissociate into CO* and OH*, further feeding the CO hydrogenation pathway. Their analysis also suggested that over-hydrogenation of CO-derived surface carbon species can lead to CH_4_ as a competing product. They further argued that the strong Cu–ZnO interaction catalyst enhanced these surface hydrogenation reactions, promoting methanol and ethanol formation and reducing CO selectivity relative to the plasma-only system.

**Fig. 8:**
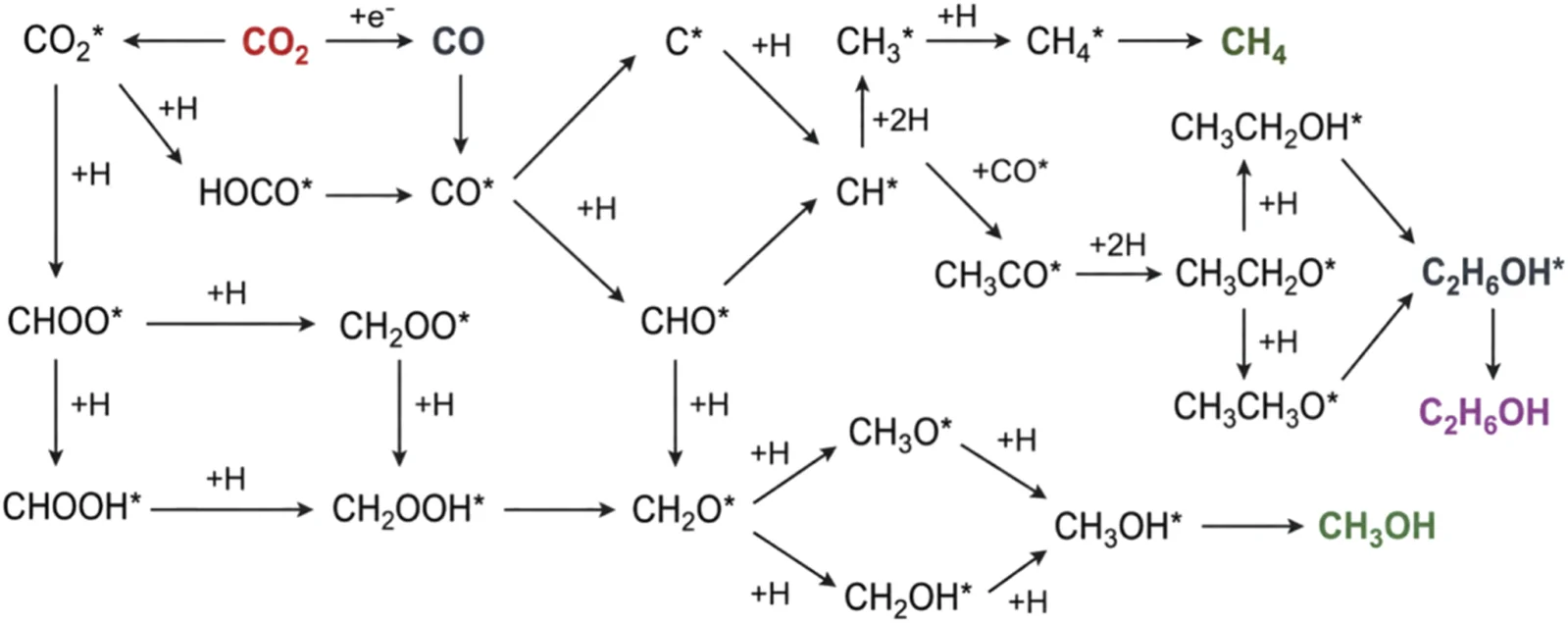
Reaction pathways for plasma-catalytic CO_2_ hydrogenation over Cu–ZnO/Al_2_O_3_ (CZA) catalysts, as proposed by Mei *et al.* Adapted from ref. 53 under the terms of the Creative Commons CC BY licence.

A related pathway was described by Pan *et al.* over H_2_/Ar plasma-treated CoO, in which stepwise CO hydrogenation operates alongside direct radical-mediated methanol formation.^49^ They proposed two pathways; Path A follows stepwise CO hydrogenation *via* CHO, CH_2_O, and CH_3_O intermediates to CH_3_OH, and Path B, which involves direct coupling of OH radicals with CH_3_ species to produce methanol. The authors further showed that oxygen vacancies on H_2_/Ar-treated CoO promoted adsorption and recombination of O-containing CH_*x*_O species, facilitating methanol synthesis through both gas-phase and surface-assisted reactions under plasma conditions.

More direct mechanistic evidence for the CO hydrogenation pathway was recently provided by Xu *et al.* using *operando* XAS, DRIFTS, and X-ray pair distribution function analysis over Cu–Zn/ZSM-5 catalysts under NTP conditions.^54^ The study demonstrated that Zn promotes Cu dispersion and stabilises Cu/ZnO interfacial sites that enable a CO hydrogenation pathway alongside the formate route on metallic Cu. Plasma enhanced gas-phase CO_2_ dissociation to CO, bypassing the RWGS step required in conventional thermal catalysis. The resulting CO adsorbed on these interfacial sites and underwent hydrogenation through HCO* intermediates towards methanol. *Operando* DRIFTS confirmed the coexistence of adsorbed CO, formyl, and formate species under plasma conditions. *Operando* XAS also showed that NTP continuously regenerated and maintained Cu in a reduced state during reaction. Cu–Zn catalysts exhibited greater resistance to re-oxidation than monometallic Cu catalysts. This was attributed to oxygen vacancies in ZnO that preferentially adsorb oxidative plasma species (*e.g.*, O* and O_2_), protecting Cu active sites.

#### 3.2.3 Carboxyl route (COOH*)

A third proposed route proceeds through a surface carboxyl intermediate (COOH*). Unlike the formate and RWGS pathways, this route has been reported primarily over bimetallic catalysts, where specific adsorption geometries stabilise the COOH* species. Wang *et al.* combined *in situ* plasma-coupled FTIR spectroscopy and DFT calculations to investigate this pathway over Ni–Co catalysts.^47^ They detected adsorbed COOH* species exclusively on the Ni–Co bimetallic catalyst. DFT calculations further showed that plasma-generated H radicals promote hydrogenation of 
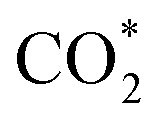
 to COOH* intermediates through an ER mechanism with minimal activation barrier. Two possible COOH* adsorption configurations were proposed (Fig. 9), although one pathway exhibited a particularly low rate-limiting energy barrier of 0.63 eV, making it the most favourable route toward methanol formation. The authors further suggested that asymmetric CO_2_ adsorption at the Ni–Co interface stabilised the carboxyl intermediates and promoted methanol synthesis over competing RWGS and CO hydrogenation pathways.

**Fig. 9:**
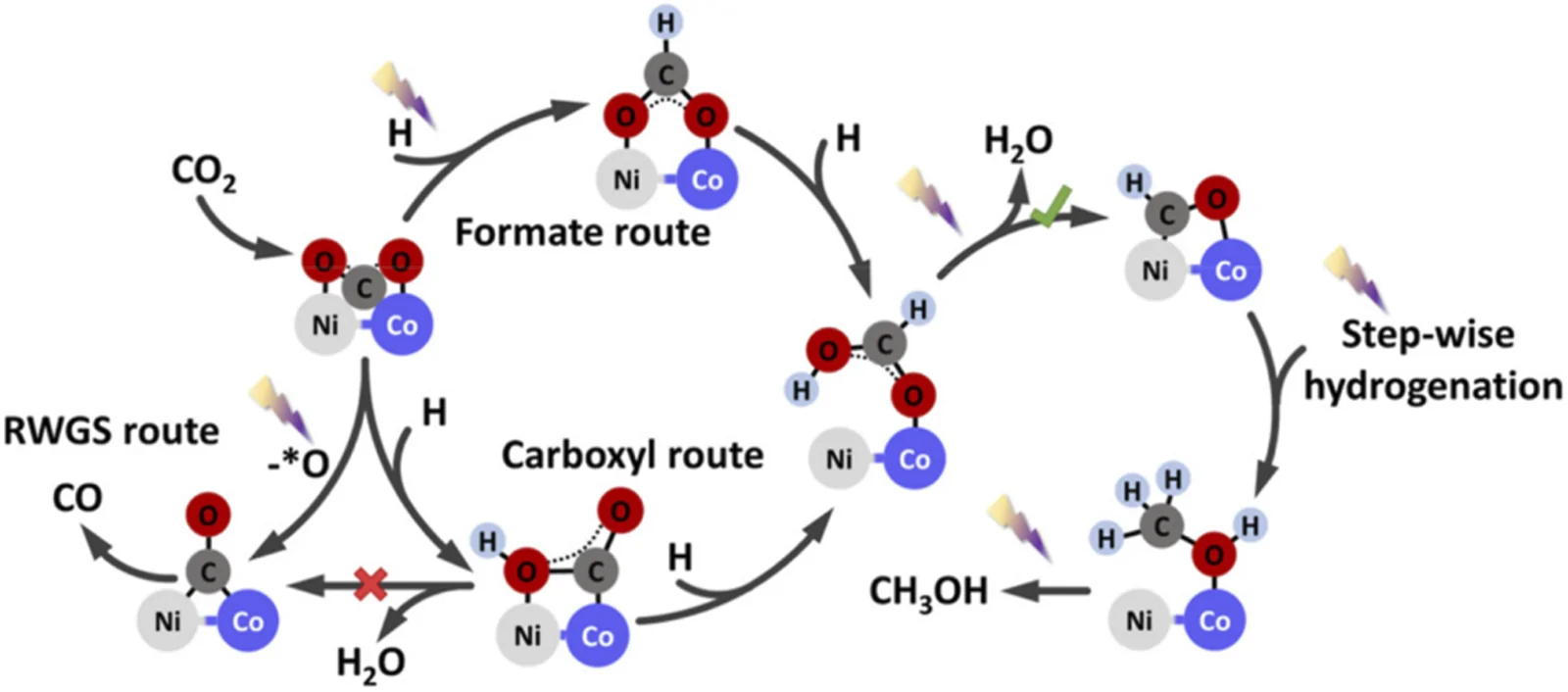
Formate, RWGS and carboxyl reaction pathways for CO_2_ hydrogenation to methanol on the Ni–Co interface, proposed by Wang *et al.* Reprinted from ref. 47 under the terms of the Creative Commons CC BY licence.

A related carboxyl mechanism was proposed by Choudhry *et al.* over Ni/Ce–Al mixed oxide, where this pathway was found to operate alongside formate and carbide routes.^46^ In this mechanism, atomic hydrogen reacts with the oxygen atom of adsorbed CO_2_ rather than the carbon atom to form adsorbed carboxyl species (COOH*). The COOH* intermediate subsequently loses oxygen to form COH*, followed by sequential hydrogenation through HCOH* and H_2_COH* intermediates before finally producing methanol.

The formate and RWGS pathways have been widely reported in plasma-assisted CO_2_ hydrogenation to methanol, but evidence for the carboxyl route remains comparatively limited. Current studies suggest that COOH* intermediates are primarily stabilised at specific catalyst interfaces, particularly bimetallic systems where unique electronic and geometric effects favour their formation. Consequently, the carboxyl pathway may depend on catalyst composition and reaction conditions rather than being a common mechanism across different catalytic systems. Further *operando* spectroscopic and theoretical studies are required to establish the contribution of this route to methanol formation.

#### 3.2.4 Carbide route

The fourth proposed route is the carbide pathway, which involves oxygen removal from adsorbed CO to form surface carbon, C*, which is then progressively hydrogenated through CH*, 
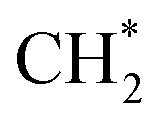
, and 
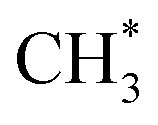
 intermediates. Methanol formation then requires incorporation of an OH group, derived from CO_2_ dissociation or surface OH species. This pathway was proposed by Choudhry *et al.* over Ni/Ce–Al mixed oxide, as shown in Fig. 10.^46^ Based on XPS evidence for carbide formation on the catalyst surface, the authors suggested that the carbide pathway likely plays a significant role in methanol synthesis under plasma conditions, alongside the formate and carboxyl routes. As previously mentioned, the carbide route may introduce a selectivity risk, since over-hydrogenation of C* beyond 
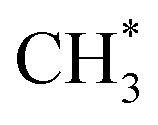
 can favour CH_4_ formation rather than methanol. Experimental and modelling studies have also reported higher methane production over catalysts that favour C–O bond cleavage, whereas stabilisation of oxygenated CH_*x*_ intermediates may help maintain methanol selectivity.^39^ However, direct evidence that carbide intermediates contribute to methanol formation remains limited, and further mechanistic studies are needed to establish their role.^46^

**Fig. 10:**
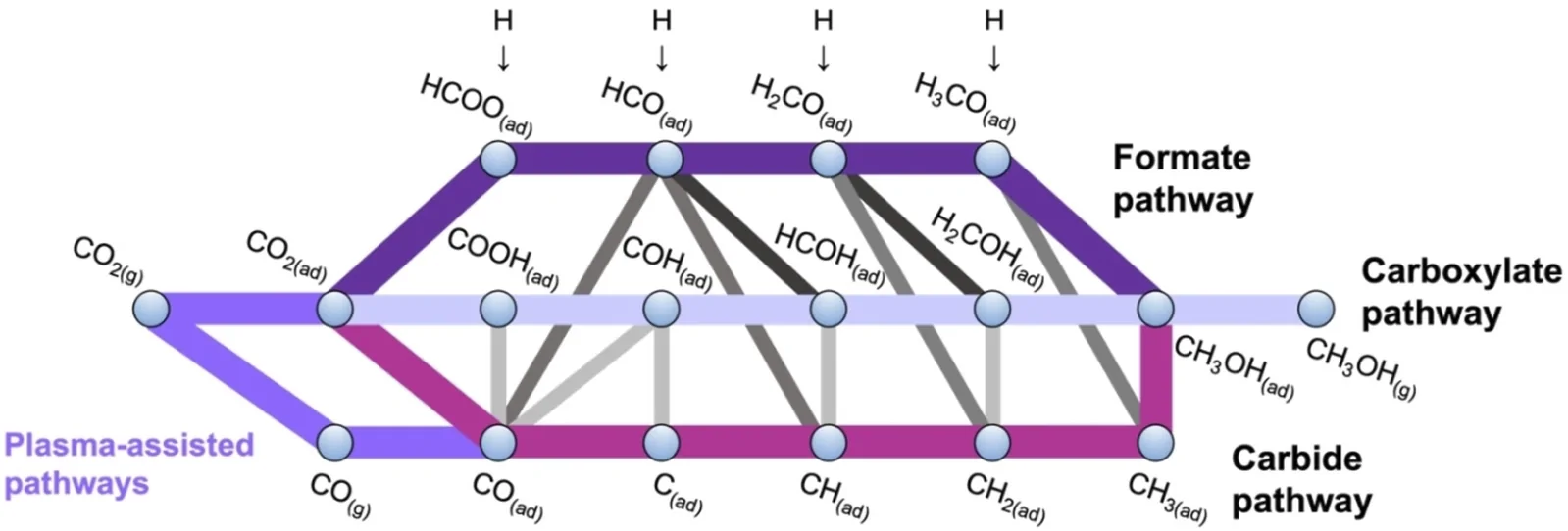
Reaction pathways for the plasma hydrogenation of CO_2_ over Ni/Ce–Al catalyst, proposed by Choudhry *et al.* Reprinted from ref. 46 under the terms of the Creative Commons CC BY licence.

### 3.3. Key mechanistic debates and cross-cutting considerations

#### 3.3.1 Plasma-specific mechanistic effects *versus* thermal analogy

A recurring issue in mechanistic interpretation is the extent to which plasma–catalytic pathways are genuinely distinct from their thermocatalytic counterparts. Many proposed surface intermediates, such as formate, methoxy, formaldehyde, and carboxyl, are also central to conventional CO_2_ hydrogenation mechanisms. Several studies acknowledge this overlap and interpret their plasma-catalytic findings within thermocatalytic frameworks. However, plasma can alter reaction pathways and kinetics through ER reactions involving gas-phase H radicals that bypass adsorption steps entirely, vibrational excitation of CO_2_ that lowers adsorption barriers, and dynamic catalyst surface modification through charging, defect generation, and oscillating oxidation states. Recent studies have attempted to explicitly separate thermal and plasma contributions by comparing plasma-catalysis, thermal catalysis, and plasma-only operation at the same reaction temperature. For example, Zeng *et al.* designed a temperature-controlled DBD reactor and demonstrated that plasma-catalytic CO_2_ hydrogenation consistently outperformed both thermal catalysis and plasma-alone modes. The authors further concluded that plasma power was more effective than heating power at the same temperature and observed substantial changes in product selectivity, indicating that plasma-generated reactive species can modify reaction pathways beyond what can be achieved through thermal activation alone. In particular, plasma excitation of CO_2_ prior to adsorption was proposed to lower activation barriers relative to conventional thermal activation of adsorbed CO_2_ species.^55^ The DFT results of Cui *et al.* illustrate the potential magnitude of these effects. Plasma-generated H radicals reduced the HCOOH* formation barrier from over 1.32 to 0.17 eV and the CH_3_OH formation barrier from 1.25 to 0.15 eV.^39^ This shows that plasma-specific activation mechanisms can fundamentally alter the kinetic landscape of surface hydrogenation steps.

#### 3.3.2 Pathway competition and co-existence

One of the most consistent findings across the literature is that no single mechanistic route operates in isolation. Studies over Cu/Al_2_O_3_, Cu–ZnO, Ni–Co, and Ni/Ce–Al systems all report multiple concurrent pathways, with relative contributions shifting depending on catalyst composition, plasma power, and other operating conditions. This raises a fundamental interpretive challenge, *i.e.*, *in situ* spectroscopic techniques such as DRIFTS can identify surface intermediates but cannot easily resolve their relative flux contributions to the observed methanol yield. Product selectivity values can provide indirect evidence of dominant pathways but unambiguously attributing them to specific upstream surface routes remains difficult when multiple pathways converge at common intermediates such as CH_3_O*.

#### 3.3.3 Discrepancies in the role of vibrational excitation

There is a notable inconsistency in the literature regarding the mechanistic importance of vibrationally excited CO_2_. Wang *et al.* acknowledged CO_2_ vibrational excitation as a potentially significant activation pathway but noted that excited species may be rapidly quenched at catalyst surfaces, rendering their contribution minor.^26^ In contrast, other modelling studies, particularly those using plasma kinetic models, attribute a more substantial role to vibrationally excited species in initiating downstream chemistry. This discrepancy likely reflects differences in reactor geometry, pressure, and discharge conditions between studies, but has not been systematically resolved. Given that vibrational excitation is often cited as a key energy-efficiency advantage of NTP, clarifying its actual mechanistic contribution under plasma-catalytic conditions is an important open question.

#### 3.3.4 Convergence at the methoxy intermediate

Despite the mechanistic diversity at early CO_2_ activation steps, there is broad agreement across many proposed methanol formation routes that methoxy (CH_3_O*) is the penultimate surface intermediate before methanol. This convergence point is significant because it suggests that the selectivity-determining step may not be the initial CO_2_ activation route. Instead, selectivity may also depend on the efficiency of methoxy hydrogenation to methanol relative to its further decomposition or competing reactions. This suggests that catalyst design targeting CH_3_O* stability and its hydrogenation kinetics could improve methanol selectivity in pathways that proceed through methoxy intermediates.

#### 3.3.5 CO retention *versus* desorption

A recurring theme across plasma-catalytic studies is what governs the branching between CO desorption as a terminal product and its further hydrogenation to methanol on the catalyst surface. In plasma-only systems, CO is the dominant carbon-containing product and methanol yields remain low. In plasma-catalytic systems, CO can be adsorbed and hydrogenated further, but CO desorption remains a competing outcome at every stage. Studies such as those by Mei *et al.* and Xu *et al.* suggest that plasma enhances gas-phase CO_2_ dissociation to CO, which then feeds the surface CO hydrogenation pathway.^53,54^ However, for catalysts with weaker CO adsorption (*e.g.* MgO, Al_2_O_3_), this CO largely desorbs rather than being hydrogenated, making catalyst identity the decisive factor. Although plasma efficiently generates CO, methanol formation largely depends on the ability of the catalyst surface to adsorb and hydrogenate CO before desorption occurs.

#### 3.3.6 Catalyst surface dynamics under plasma

Several studies highlight that plasma actively modifies catalyst surface oxidation state during reaction, for example, stabilising reduced Cu species,^54^ or dynamic reduction–oxidation of Fe species and oxygen vacancies.^52^ These dynamic surface changes are rarely accounted for in static DFT or microkinetic models, which typically assume a fixed surface structure. The implication is that the active catalyst surface under plasma-catalytic conditions may differ substantially from its pre-reaction or post-reaction characterisation, introducing uncertainty into mechanistic interpretations that rely on *ex situ* or even steady-state *in situ* characterisation.

## 4. Catalyst design for NTP CO_2_ hydrogenation to methanol

The mechanistic analysis presented in the previous section established that catalyst composition is a primary determinant of which hydrogenation pathway dominates in plasma-catalytic systems. However, the choice of active metal alone is insufficient to explain the wide variation in methanol yield and selectivity reported across studies. Surface and structural features, including basicity, support porosity, metal dispersion, oxygen vacancy density, and metal–support interactions, exert strong influence on plasma discharge behaviour and catalytic performance.^56^ As a general design principle, the oxygen adsorption energy (Δ*E*_O_) of surface intermediates serves as a useful descriptor. If oxygen binds too weakly, intermediates are unstable and reaction barriers remain high, and if it binds too strongly, it leads to surface poisoning. An intermediate Δ*E*_O_ is therefore optimal because it stabilises key intermediates and still allows further hydrogenation and desorption.^57^ Early plasma-catalytic studies using Cu/ZnO/Al_2_O_3_ under high-pressure, high-power discharge conditions reported methanol selectivity below 2%, demonstrating that industrial thermal catalyst formulations do not automatically translate to good plasma-catalytic performance.^58^ This highlights that catalyst design must be specifically tailored to the plasma environment, where reactive species, surface charging, and non-equilibrium conditions create a fundamentally different catalytic landscape compared to conventional thermocatalysis. The following sections survey progress in monometallic and bimetallic catalyst design, organised by active metal, with attention to the structure–activity relationships that govern plasma-catalytic methanol selectivity.

### 4.1. Monometallic catalysts

#### 4.1.1 Copper

Cu-based catalysts have attracted the most attention for plasma-catalytic CO_2_ hydrogenation to methanol because copper is already well established in conventional thermocatalytic methanol synthesis and possesses a theoretically favourable Δ*E*_O_. Volcano-plot analysis by Studt *et al.* showed that metallic Cu sits closest to the activity peak under atmospheric conditions because it exhibits intermediate binding strength toward CO_2_-hydrogenation intermediates.^57^ This provides a thermodynamic rationale for its widespread use.^26^ Cu catalysts combined with promoters such as Zn, Zr, Ce, Ga, and Cr and supported on materials including Al_2_O_3_, ZnO, ZrO_2_, SiO_2_, Nb_2_O_5_, and carbon-based supports have been reported to enhance CO_2_ hydrogenation performance through improved CO_2_ activation, stabilisation of key reaction intermediates, higher methanol selectivity, and increased catalytic stability. In plasma-catalytic systems, the Cu-support interface can further create bifunctional active sites that simultaneously promote CO_2_ activation and hydrogenation, resulting in higher CO_2_ conversion and methanol yield.^26,39^

Cui *et al.* investigated CO_2_ hydrogenation over Cu/γ-Al_2_O_3_ in a packed-bed coaxial DBD reactor operated at atmospheric pressure with a CO_2_ : H_2_ ratio of 1 : 3, a total flow rate of 72 mL min^−1^, an input power of 26 W, and a discharge frequency of 9.5 kHz.^39^ Relative to plasma alone, the introduction of γ-Al_2_O_3_ packing and subsequently Cu/γ-Al_2_O_3_ significantly enhanced CO_2_ conversion and methanol selectivity, reaching selectivities above 60% under optimised H_2_-rich conditions. DFT calculations showed that Cu–Al_2_O_3_ interface plays a dual role by simultaneously activating CO_2_ and stabilising hydrogenation intermediates.

Wang *et al.* compared Cu/γ-Al_2_O_3_ and Pt/γ-Al_2_O_3_ catalysts in an atmospheric-pressure, DBD reactor operated at a discharge power of 10 W and frequency of 9 kHz using a CO_2_ : H_2_ ratio of 1 : 3 and a total gas flow rate of 40 mL min^−1^. The reactor employed a 45 mm discharge length and a 3 mm discharge gap, while the water-cooled electrode maintained the reaction temperature at approximately 30 °C.^26^ Cu/γ-Al_2_O_3_ showed superior methanol synthesis performance, achieving a maximum methanol yield of 11.3%, methanol selectivity of 53.7%, and CO_2_ conversion of 21.2%. No measurable reaction occurred under the same conditions without plasma, confirming that catalytic activity in this system is entirely plasma dependent. The superior performance of Cu was attributed to its favourable interaction with plasma-generated reactive species.

Support basicity and metal–support interactions play a critical role in determining methanol selectivity in Cu-based systems. Chen *et al.* investigated Cu_*x*_O/MgO catalysts in a packed-bed coaxial DBD reactor operated at atmospheric pressure and 30 °C using a CO_2_ : H_2_ ratio of 1 : 3, a total flow rate of 72 mL min^−1^, a discharge frequency of 9.2 kHz, and a discharge power of 24 W. Under these conditions, the Cu_*x*_O/MgO catalyst, made *via* a sol–gel method using MgSO_4_ and KOH as the precursor and gelling agent, respectively, achieved 9.1% CO_2_ conversion with 46.2% methanol selectivity.^59^ Strong interaction between Cu_*x*_O species and defective low-crystallinity MgO increased Cu dispersion and generated abundant Cu–MgO interfacial sites that promoted sequential hydrogenation toward methanol. Surface adsorbed oxygen species also correlated positively with methanol formation. The catalyst favoured formate-route hydrogenation and suppressed CO formation and deep hydrogenation pathways and interestingly showed no significant decline in CO_2_ conversion or methanol selectivity over a 24 hours continuous plasma-catalytic reaction.

The importance of Cu(i) species under plasma conditions was highlighted by Zou *et al.*^60^ They used a DBD plasma reduction treatment to convert Cu(ii) sites in HKUST-1 into Cu(i) under near-ambient conditions, using an input power of 35 W, a total flow rate of 30 mL min^−1^ and a CO_2_ : H_2_ ratio of 1 : 3. The treatment preserved the MOF framework structure. Plasma activation enabled efficient valence modulation at 40 °C and avoided the severe agglomeration and structural collapse often observed during conventional thermal reduction of Cu-based materials. XPS analysis showed that CO plasma treatment generated catalysts containing up to 76.5% Cu(i). *In situ* DRIFTS analysis suggested that plasma-excited reducing species promoted rapid Cu(ii) reduction through strong adsorption on copper sites. The Cu(i)-rich catalyst showed enhanced oxygenate formation during plasma-assisted CO_2_ hydrogenation. The results support the proposed role of Cu(i) species in stabilising oxygenated intermediates during methanol synthesis.

#### 4.1.2 Cobalt

Certain catalysts such as those based on cobalt, if not optimised properly, can promote CO_2_ hydrogenation toward a wide range of products including methanol, CO, methane, and higher hydrocarbons.^43^ This highlights that achieving high methanol selectivity requires careful control of catalyst structure and plasma conditions. Ronda-Lloret *et al.* investigated Co_*x*_O_*y*_/MgO catalysts in a water-cooled coaxial DBD reactor operated at atmospheric pressure and 35 °C with a discharge power of 10 W, a frequency of 9.2 kHz, a CO_2_ : H_2_ ratio of 1 : 3, and a total gas flow rate of 28 mL min^−1^. Under these conditions, methanol formation was strongly dependent on cobalt loading.^43^ As shown in Fig. 11, a low loading of 5 wt% gave moderate CO_2_ conversion (∼30%) but produced mainly CO with limited methanol formation. Increasing the loading to 15 wt% improved the dispersion of Co_*x*_O_*y*_ species and increased the density of metal–support interfacial sites, raising methanol selectivity to 31% at 33% CO_2_ conversion. However, further increasing the loading to 20 wt% caused particle growth and partial pore blockage, reducing catalytic performance. The support also played a critical role. Basic MgO outperformed acidic γ-Al_2_O_3_ by enhancing CO_2_ adsorption and improving methanol selectivity under plasma conditions. The catalyst maintained stable performance for up to 4.5 h, with minimal deactivation.

**Fig. 11:**
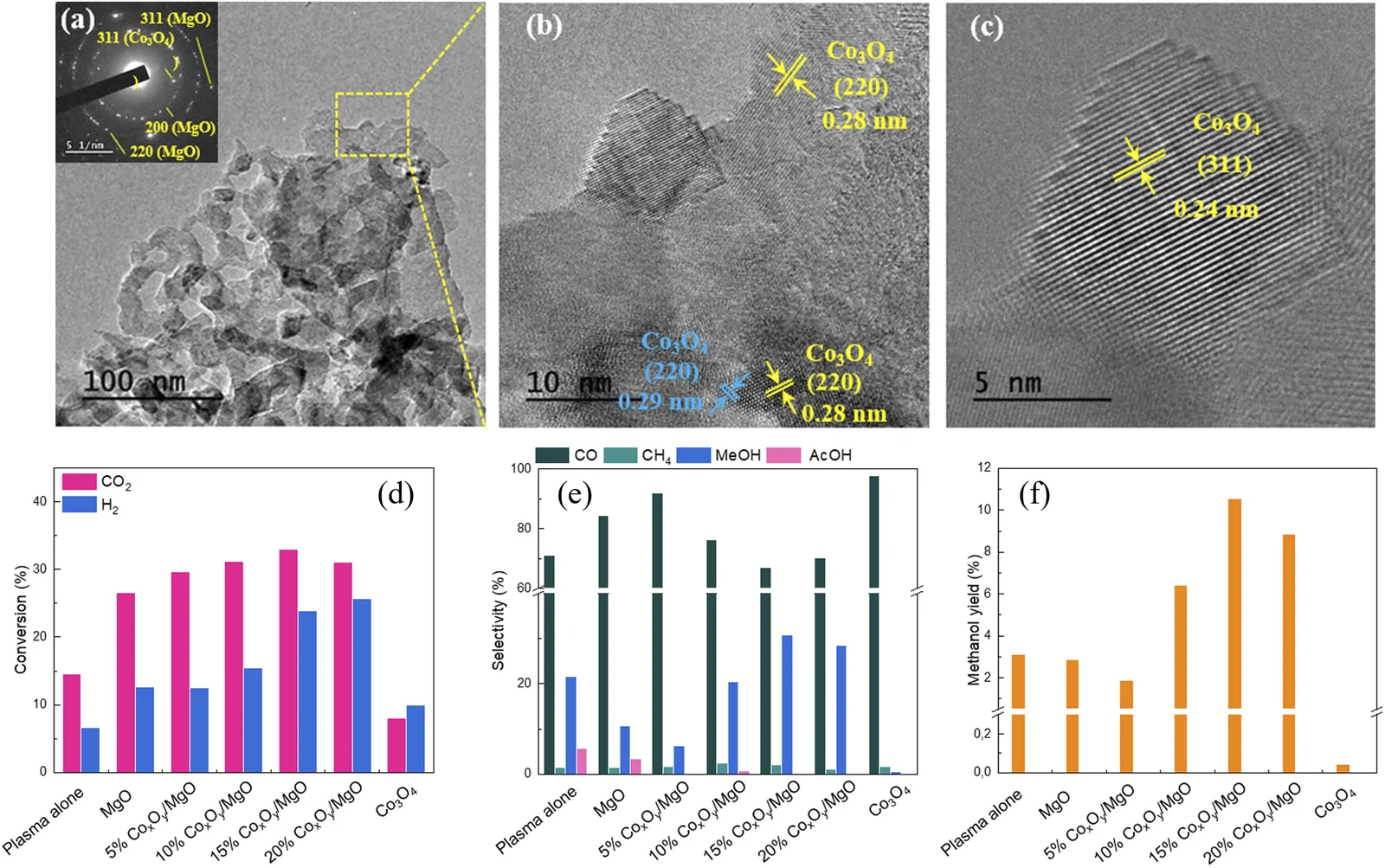
Representative TEM images of 15% Co_*x*_O_*y*_/MgO made by Ronda-Lloret *et al.* (a) TEM micrograph and its corresponding SAED pattern (inset), (b and c) magnified HRTEM images of 15% Co_*x*_O_*y*_/MgO, (d) CO_2_ and H_2_ conversion, (e) methanol selectivity, and (f) methanol yield for plasma alone, MgO, and Co_*x*_O_*y*_/MgO with different cobalt metal loadings, and unsupported Co_3_O_4_. Reaction conditions: 35 °C, atmospheric pressure, 500 mg catalyst, total flow rate = 28 mL min^−1^, CO_2_ : H_2_ = 1 : 3, discharge power = 10 W, and reaction time = 1.5 h. Adapted from ref. 43 under the terms of the Creative Commons CC BY licence.

The role of surface hydrophobicity was demonstrated by Pan *et al.*,^48^ using a physical mixture of Co_3_O_4_/Al_2_O_3_ (5.1 wt% Co) and PTFE in a fluidised-bed coaxial DBD reactor operated at atmospheric pressure (0.1 MPa), 25 °C, using a 4 kV, 18 kHz AC power supply, and a total gas flow rate of 800 mL min^−1^ (CO_2_ : H_2_ : Ar = 25 : 75 : 700 mL min^−1^). The PTFE-modified catalyst achieved 17.5% CO_2_ conversion with 66.2% methanol selectivity, under ambient conditions, substantially outperforming the unmodified catalyst (Fig. 12). The improvement was attributed to increased surface hydrophobicity, which accelerated H_2_O desorption and reduced competitive adsorption of water on catalytic sites. This maintained greater availability of active sites for CO_2_ hydrogenation and enhanced the formation and consumption of oxygenated intermediates, particularly CH_3_O*. Kinetic analysis showed that the CH_3_O* reaction rate increased by nine orders of magnitude after PTFE incorporation. Interestingly, PTFE underwent some limited surface degradation during plasma exposure, but the composite structure and hydrophobic functionality remained sufficiently stable to maintain catalytic performance during their stability tests. This study highlights the importance of surface microenvironment engineering in plasma-catalytic methanol synthesis.

**Fig. 12:**
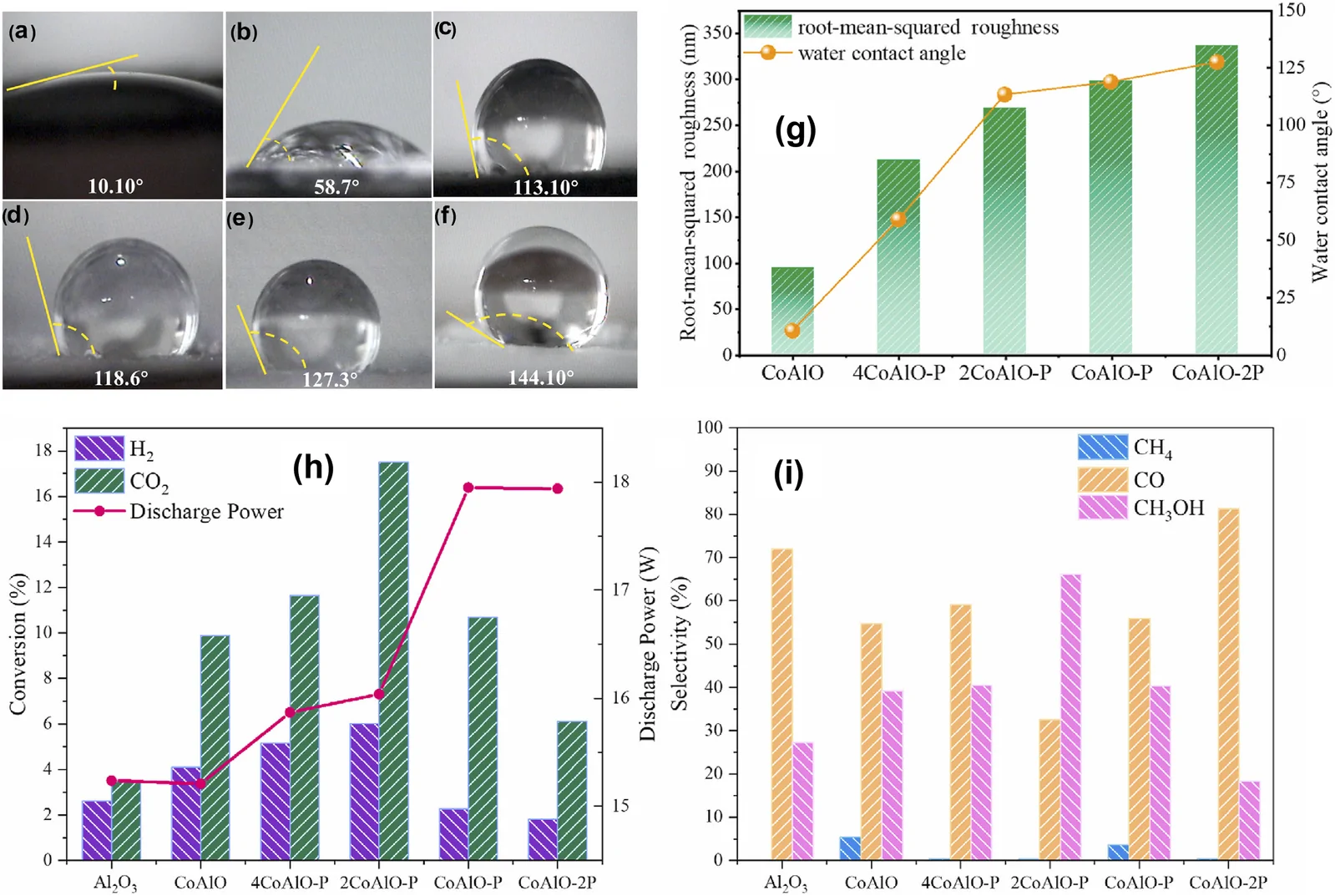
(a–f) Water contact angle measurements, (g) relationship curve between roughness and water contact angle, (h) H_2_ and CO_2_ conversion rates and discharge power; (i) selectivity toward CH_4_, CO, and CH_3_OH, for PTFE-modified Co_3_O_4_/Al_2_O_3_ catalysts developed by Pan *et al.* Reaction conditions: total gas flow rate = 800 mL min^−1^, Ar: 700 mL min^−1^, H_2_: 75 mL min^−1^, CO_2_: 25 mL min^−1^; AC power supply: 4 kV, 18 kHz. Adapted from ref. 48 with permission from Elsevier, copyright 2026.

Pan *et al.* synthesised CoO-based catalysts and subsequently applied N_2_ or H_2_/Ar plasma pretreatments before catalytic testing to generate oxygen-vacancy-rich surfaces. The resulting H_2_/Ar-plasma-treated CoO catalyst (H_2_/Ar–CoO) exhibited a CH_3_OH selectivity of 39.6%, approximately four times higher than that of untreated CoO (10.0%) under optimised conditions of 11 kV and 7 kHz.^49^ The improvement was attributed to Ar^+^ bombardment and H-assisted reduction during plasma treatment, which increased OH and CH_3_O species. H_2_O addition supplied OH and H radicals through electron-impact dissociation, promoting CH_3_O + H and CH_3_ + OH recombination pathways toward methanol. However, they also argued that H_2_O can consume energetic electrons and slightly reduce CO_2_ conversion, so its benefit is mainly associated with enhanced methanol selectivity and energy utilisation rather than conversion alone. Furthermore, the authors highlighted the importance of oxygen-vacancy stability in sustaining plasma-catalytic activity. The H_2_/Ar plasma-treated CoO catalyst retained its oxygen-vacancy-rich structure during catalytic testing and maintained enhanced methanol selectivity relative to untreated CoO.

#### 4.1.3 Nickel

Nickel-containing mixed oxides can achieve competitive methanol selectivity under plasma conditions when combined with reducible oxide supports. Choudhry *et al.* investigated Ni/Ce–Al mixed oxide (MOx) catalysts in a packed-bed DBD reactor operated at atmospheric pressure using a sinusoidal voltage of 10 kV at 10 kHz. The reactor had a discharge length of 35 mm and a discharge gap of 1.5 mm and was fed with CO_2_ : H_2_ mixtures at a total flow rate of 52 sccm under near-ambient conditions (∼30 °C).^46^ They found that Ni/Ce–Al mixed oxide gave the best performance, reaching 19.9% CO_2_ conversion and 66.2% methanol selectivity under optimised conditions (Fig. 13). This substantially outperformed Ni/CeO_*x*_, Ce–Al MO_*x*_, and the no-catalyst baseline. The superior performance was attributed to the complementary roles of Ni in H_2_ activation and CeO_2_ in CO_2_ adsorption and oxygen-vacancy formation, with the high Ce^3+^ surface concentration indicating abundant defect sites. The mixed oxide support was also proposed to enhance O^2−^ mobility and plasma–surface interactions through its relatively high ionic conductivity. Post-reaction XPS showed carbide species on the catalyst surface, suggesting that a carbide route may operate alongside formate and carboxyl pathways. However, the authors emphasised that these pathways remain postulated and require *operando* validation. Unlike Cu-based catalysts, which are typically associated with formate-mediated methanol synthesis, Ni-containing systems appear more capable of stabilising alternative pathways involving carboxylate and carbide intermediates under plasma conditions. The strong hydrogenation activity of Ni also increases the risk of over-hydrogenation toward methane, meaning that support composition and oxygen-vacancy density become critical for controlling product selectivity. The stability of the Ni/Ce–Al MO_*x*_ catalyst was assessed through three consecutive activation–reaction cycles, each preceded by reduction at 600 °C for 2 h and Ar plasma treatment for 3 h. After three cycles a cumulative reduction of 3.5% in methanol yield was reported.

**Fig. 13:**
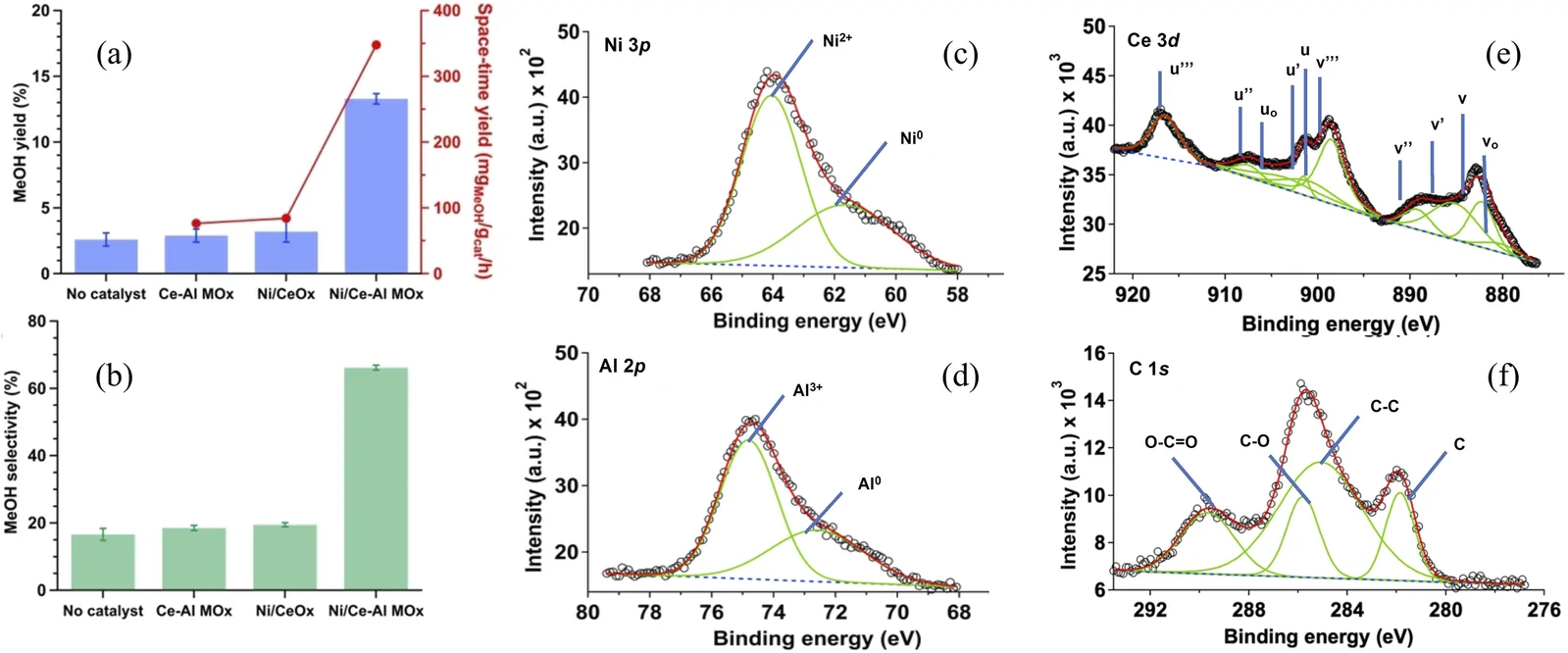
Performance of Ni/Ce–Al MO_*x*_ catalysts made by Choudhry *et al.* in plasma-assisted hydrogenation of CO_2_. (a) Methanol yield and space-time yield, (b) methanol selectivity, as well as XP spectra of Ni/Ce–Al MO_*x*_ catalyst after the reaction: (c) Ni 3p, (d) Al 2p, (e) Ce 3d, and (f) C 1s. Reaction condition: feed gas: 39 sccm H_2_ and 13 sccm CO_2_; coolant temperature: 13 °C; and applied sinusoidal voltage: 10 kV at 10 kHz. Adapted from ref. 46 under the terms of the Creative Commons CC BY licence.

#### 4.1.4 Manganese

Manganese oxide catalysts have received comparatively limited attention for methanol synthesis most likely because MnO_*x*_ alone shows poor activity under conventional thermal conditions. Zhang *et al.* demonstrated that coupling MnO_*x*_/ZrO_2_ with DBD plasma fundamentally changes this behaviour.^40^ Under optimised conditions (CO_2_ : H_2_ = 1 : 4, total flow rate = 30 mL min^−1^, discharge power = 5 W), they achieved a CO_2_ conversion of 11%, 10% methanol selectivity, and a methanol space–time yield (STY) of 0.17 mmol g_cat_^−1^ h^−1^. To quantify the non-additive interaction between plasma and catalyst, the authors introduced a plasma-catalyst synergy factor, calculated as the methanol selectivity achieved in the plasma-catalytic system divided by the sum of the selectivities obtained in the plasma-only and catalyst-only systems. Using this definition, a high synergy factor of 87 was reported, indicating that methanol formation primarily originated from the cooperative interaction between plasma-generated species and the MnO_*x*_/ZrO_2_ catalyst rather than from either component acting independently. *In situ* DRIFTS showed that plasma shifted the dominant surface intermediates from thermally favoured (bi)carbonate species to HCOO* and CH_3_O* intermediates. Plasma-generated species, including 
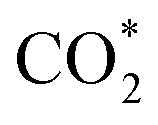
, CO_2_^+^ and H radicals, were proposed to promote HCOO* formation. MnO_*x*_ promoted the subsequent hydrogenation of HCOO* toward CH_3_O*. XPS analysis further indicated that plasma reduction increased the Mn^2+^ content, which the authors associated with enhanced methanol formation. The stability of the catalyst was tested for five consecutive cycles without regeneration, each separated by ∼10 h. CO_2_ conversion remained steady at 10% throughout the 15 hours runs, whereas methanol selectivity started at 10%, decreased slightly, and stabilised near 9%. This minor decline was attributed to gradual temperature rise (up to ∼90 °C) during prolonged discharge. Importantly, selectivity recovered at the start of each cycle, indicating that the system maintained good stability and could regain initial activity without regeneration.

#### 4.1.5 Iron

Iron-based catalysts are attractive for plasma-catalytic CO_2_ hydrogenation because of their redox flexibility, strong CO adsorption capacity, and ability to generate oxygen-deficient surface species that can participate in RWGS and methanol synthesis pathways. Meng *et al.* investigated Fe_2_O_3_/γ-Al_2_O_3_ catalysts in a packed-bed DBD reactor operated at atmospheric pressure with a 50 mm discharge length, a 4 mm discharge gap, a discharge frequency of 9 kHz, and an effective discharge power of 18 W. The feed consisted of CO_2_ and H_2_ at flow rates of 18 and 57 mL min^−1^, respectively, and the catalyst-bed temperature was approximately 80 °C.^52^ Among a series of metal oxide catalysts evaluated under mild plasma conditions, Fe_2_O_3_ supported on γ-Al_2_O_3_ was identified as the best performer, achieving 12% CO_2_ conversion and 58% methanol selectivity at 80 °C (Fig. 14). The optimal Fe loading was 5 wt%. XPS analysis showed that the fraction of chemisorbed oxygen species (O_β_) correlated with both CO_2_ conversion and methanol selectivity. CO_2_-DRIFTS, CO_2_-TPD, and CO-TPD further indicated that stronger CO_2_ and CO adsorption over Fe_2_O_3_/γ-Al_2_O_3_ contributed to improved performance. In addition, the Fe_2_O_3_/γ-Al_2_O_3_ catalyst reduced the energy consumption for methanol production from 181.7 kJ mmol^−1^ in the plasma-only system to 19.8 kJ mmol^−1^, indicating a strong plasma-catalyst synergy. Table 2 provides an overview of monometallic catalysts investigated for plasma-catalytic CO_2_ hydrogenation to methanol.

**Fig. 14:**
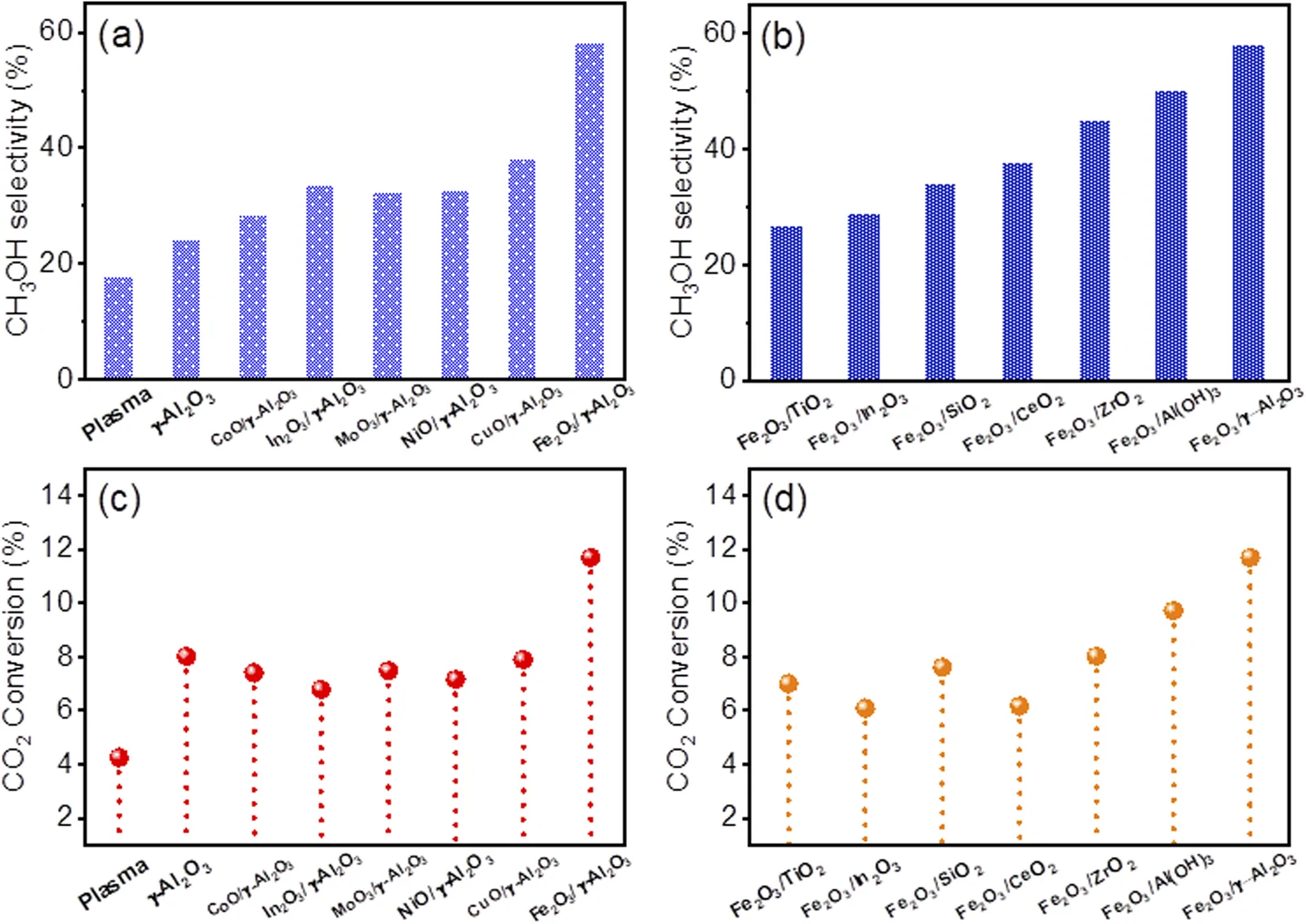
Effect of catalyst composition on its performance during plasma-catalytic CO_2_ hydrogenation. (a and c) Influence of the active metal supported on γ-Al_2_O_3_ on methanol selectivity and CO_2_ conversion, respectively. (b and d) Influence of the support type on methanol selectivity and CO_2_ conversion over Fe_2_O_3_-based catalysts, respectively. Reaction conditions: discharge power = 18 W, 9 kHz, CO_2_ : H_2_ = 1 : 3, residence time = 3.1 s, circulating water temperature = 60 °C. Reprinted from electronic supplementary information of ref. 52 with permission from Wiley, copyright 2023.

**Table 2:** Summary of monometallic catalysts reported for plasma-catalytic CO_2_ hydrogenation to methanol

Catalyst	Temp. (°C)	SEI a (kJ L^−1^)	CO_2_ : H_2_	WHSV b (mL g^−1^ h^−1^)	*X*_CO_2__ c (%)	S_MeOH_^d^ (%)	MeOH STY e (mmol g_cat_^−1^ h^−1^)	MeOH EE f (mmol kWh^−1^)	Ref.
Ni/Ce–Al	13	43.3	1 : 3	3120	19.9	66.2	10.9	—	46
Co_*x*_O_*y*_/MgO	35	21.4	1 : 3	3360	33.0	31.0	3.8	190.8	43
Fe_2_O_3_/γ-Al_2_O_3_	60	14.4	1 : 3	3750	12.0	58.0	2.8	181.8	52
Fe_2_O_3_/Al(OH)_3_	60	14.4	1 : 3	3750	9.6	50.0	1.9	—	52
Fe_2_O_3_/ZrO_2_	60	14.4	1 : 3	3750	8.0	44.5	1.4	—	52
Cu_*x*_O/MgO	30	20	1 : 3	2880	9.1	46.2	1.4	—	59
Cu/γ-Al_2_O_3_	Ambient	21.7	1 : 3	2400	10.0	48.0	1.3	86.5	39
CuO/γ-Al_2_O_3_	60	14.4	1 : 3	3750	8.0	37.5	1.2	80.2	52
Cu/γ-Al_2_O_3_	30	15	1 : 3	800	21.0	53.7	1.0	306	26
MgO	35	21.4	1 : 3	3360	27.0	10.0	1.0	50	43
Fe_2_O_3_/SiO_2_	60	14.4	1 : 3	3750	7.5	33.4	1.0	—	52
In_2_O_3_/γ-Al_2_O_3_	60	14.4	1 : 3	3750	6.9	33.5	0.9	60.8	52
Cu(i)-HKUST	40	70	1 : 3	3600	33.4	6.9	0.9	—	60
Fe_2_O_3_/CeO_2_	60	14.4	1 : 3	3750	6.1	36.7	0.9	—	52
Pt/γ-Al_2_O_3_	30	15	1 : 3	800	17.5	51.4	0.8	240	26
MnO_*x*_/ZrO_2_	Ambient	7.5	1 : 4	2400	11.0	10.0	0.2	—	40
Co_3_O_4_	35	21.4	1 : 3	3360	8.0	—	—	—	43
Co_3_O_4_/Al_2_O_3_–PTFE	25	1.2	1 : 3^g^	—	17.5	66.2	—	486	48
CoO (dry feed)	10	27	1 : 3	—	4.3	30.0	—	—	49
CoO (water co-fed)	10	27	1 : 3	—	4.5	39.6	—	—	49

a Specific energy input.

b Weight hourly space velocity.

c CO_2_ conversion.

d Methanol selectivity.

e Methanol space-time yield.

f Energy efficiency.

g CO_2_ : H_2_ : Ar = 1 : 3 : 28.

### 4.2. Bimetallic and multicomponent catalysts

Monometallic catalysts often exhibit limitations in plasma-catalytic methanol synthesis due to the complexity of the competing reaction pathways. Cu-based systems, for example, can suffer from HCOO* accumulation caused by insufficient further hydrogenation, agglomeration of active Cu species, and competition from RWGS that lowers methanol selectivity. Bimetallic catalysts can alleviate these limitations by modifying the electronic structure of the active phase, enhancing H_2_ activation, promoting hydrogen spillover, and generating additional interfacial active sites. Insights from conventional thermal methanol synthesis have also motivated this approach. Ni–Ga and Pd–Ga alloys, for example, have shown promising activity as alternatives to Cu-based catalysts under low-pressure thermal conditions.^13^ These concepts have subsequently inspired the development of plasma-catalytic bimetallic systems designed to improve methanol selectivity.

Cu–Zn systems have shown beneficial bimetallic interactions under plasma conditions. Chen *et al.* demonstrated this using Cu–Zn catalysts supported on mesocellular foam (MCF) silica in a packed-bed DBD reactor operated at ambient temperature and pressure with a feed composition of 20 vol% CO_2_, 60 vol% H_2_, and 20 vol% Ar (50 mL min^−1^ total flow rate), an applied peak voltage of 10.1 ± 0.4 kV, a frequency of 7.7 kHz, and a discharge power of 20.9 ± 1.3 W.^56^ Introducing 1 wt% Cu onto MCF increased the methanol STY to 0.225 mmol g_cat_^−1^ h^−1^ and suppressed CH_4_ formation relative to bare MCF packing. Addition of Zn as a promoter created strong Cu–Zn interactions that further enhanced CO_2_ and CO adsorption and improved methanol synthesis performance. The Cu1Zn1/MCF catalyst achieved the highest methanol STY (0.275 mmol g_cat_^−1^ h^−1^), reduced the energy consumption to 26.4 kJ mmol_MeOH_^−1^, and maintained stable operation over 40 h. The improved performance was attributed to synergistic interactions between Cu and Zn species, which promoted adsorption and stabilisation of key oxygenated intermediates. The study also showed that excessive Cu loading (>1 wt%) reduced performance, indicating that maintaining high metal dispersion remains critical in plasma-catalytic systems.

Chen *et al.* introduced Beta zeolite as a tertiary component in a CuO–MgO/Beta zeolite catalyst and evaluated it in a packed-bed coaxial DBD reactor operated at atmospheric pressure (0.1 MPa) and 30 °C. The reactor employed a 60 mm discharge length packed with 1.5 g catalyst, a discharge frequency of 9.2 kHz, a discharge power of 24 W, and a CO_2_ : H_2_ feed ratio of 1 : 3 (18 and 54 mL min^−1^, respectively). Under these conditions, the catalyst achieved 8.5% CO_2_ conversion and 72% methanol selectivity under ambient conditions.^41^ Strong CuO–MgO interactions modified the electronic structure of Cu species and facilitated the formation of active Cu_2_O-containing sites. MgO basic sites enhanced CO_2_ adsorption as carbonate species and promoted subsequent hydrogenation through the formate pathway, suppressing RWGS-derived CO formation. The combined CuO–MgO synergy and porous Beta zeolite support produced one of the highest methanol selectivities reported for Cu-based plasma-catalytic systems. The optimised catalyst (10 wt% Cu and 15 wt% Mg) maintained consistent performance for 24 h.

Cu–ZnO synergy, which is well established in thermal methanol synthesis, has also shown beneficial effects under plasma conditions. Mei *et al.* investigated Cu–ZnO/Al_2_O_3_ foam ceramic catalysts in a parallel-plate DBD reactor operated at atmospheric pressure with an AC power supply, a discharge frequency of 10 kHz, a discharge power of 25 W, a CO_2_ : H_2_ ratio of 1 : 3, and a total flow rate of 40 mL min^−1^. The reactor consisted of two parallel quartz plates separated by a 5 mm discharge gap and packed with catalyst. Under these conditions, the catalyst-bed temperature stabilised at approximately 190 °C.^53^ They found that the CuO : ZnO mass ratio of 2 : 1 (CZA21) delivered the best performance. The catalyst achieved 22.4% CO_2_ conversion, 15.5% H_2_ conversion, and 20.9% methanol selectivity. The improved activity was attributed primarily to strong Cu–ZnO interactions that enhanced Cu dispersion, reduced Cu particle size, and increased the concentration of strong basic sites involved in CO_2_ adsorption and activation. The CZA21 catalyst also exhibited the largest specific surface area among the tested compositions, which further contributed to its superior performance.

Cu–Ni bimetallic catalysts have also shown improved methanol synthesis performance relative to monometallic Cu systems. Chen *et al.* identified Cu–Ni/γ-Al_2_O_3_–CeO_2_ as the best-performing catalyst among several Cu-based formulations in a packed-bed coaxial DBD reactor operated at atmospheric pressure (0.1 MPa) and 150 °C. The reactor employed an effective discharge length of 100 mm, was packed with 6 g catalyst (20–40 mesh), and operated at a specific energy input (SEI) of 21 J cm^−3^ with a weight hourly space velocity (WHSV) of 19 200 mL g_cat_^−1^ h^−1^.^51^ The catalyst achieved 27.5% CO_2_ conversion, 34.7% methanol selectivity. This represented a substantial improvement over plasma-only operation, increasing the methanol STY from 0.19 to 2.06 mmol g_cat_^−1^ h^−1^ and reducing the energy consumption from 385.03 to 34.91 kJ mmol^−1^. The improved performance was attributed primarily to Cu–Ni bimetallic interactions that enhanced Cu dispersion and promoted H_2_ activation through hydrogen spillover. These effects accelerated hydrogenation of oxygenated intermediates toward methanol and suppressed CO formation. CeO_2_ further improved CO_2_ adsorption and activation through oxygen vacancies and weak basic sites. The optimised catalyst maintained its performance over a 48 hours run.

Cu–Co bimetallic catalysts have also shown clear performance improvements over their monometallic counterparts in plasma-catalytic methanol synthesis. Fang *et al.* prepared SiO_2_-supported Cu–Co catalysts with different Cu : Co mass ratios and evaluated them in an atmospheric-pressure, water-cooled DBD reactor operated at 29 °C, 10.2 kV, 7.7 kHz, 11 W (SEI = 13.2 kJ L^−1^), using a feed of 20 vol% CO_2_, 60 vol% H_2_, and 20 vol% Ar at a total flow rate of 50 mL min^−1^.^42^ Among them, Cu7Co3–300 (Cu : Co mass ratio = 7 : 3, reduced at 300 °C) delivered the best performance. The catalyst achieved 15.3% CO_2_ conversion and a methanol STY of 0.081 mmol g_cat_^−1^ h^−1^, nearly twice that of the monometallic Cu and Co catalysts. Although the authors reported 18.1% methanol selectivity on a CO-free basis, including CO gives an estimated carbon-based selectivity of approximately 0.4%, indicating that CO remained the dominant product. The improved methanol productivity was attributed to strong interfacial interactions between metallic Cu and Co_3_O_4_. Electron transfer from Cu to Co_3_O_4_ weakened H adsorption on Cu and strengthened it on Co_3_O_4_, allowing Co_3_O_4_ to act as a hydrogen reservoir through hydrogen spillover. This promoted CO hydrogenation on Cu sites and HCOO* hydrogenation on Co_3_O_4_ sites, leading to improved HCOO* consumption and enhanced methanol formation. Reduction at 300 °C produced the most favourable structure by minimising crystallite size and improving dispersion of the active phases.

Joshi and Loganathan investigated CuO/Fe_2_O_3_ and NiO/Fe_2_O_3_ catalytic systems supported on quartz wool (QW) under NTP surface-discharge conditions.^61^ Among the tested catalysts, CuO/Fe_2_O_3_/QW exhibited the highest productivity, achieving 16.7% CO_2_ conversion and 32.7% methanol selectivity at 200 °C with 2 W plasma input power. The methanol STY reached 9.32 mmol g_cat_^−1^ h^−1^, exceeding several reported plasma-catalytic systems. The superior performance of the Cu–Fe catalyst was attributed to synergistic interactions between Cu and Fe oxide phases, which improved catalyst reducibility and facilitated CO_2_ activation and hydrogenation. In contrast, Ni-containing systems showed greater tendency toward methane formation, indicating that the balance between hydrogenation activity and over-hydrogenation remains highly sensitive to metal composition under plasma conditions.

Apart from Cu-based catalysts, Ni–Co catalysts have also shown strong plasma-catalytic methanol synthesis performance thanks to strong interfacial effects that promote the carboxyl pathway. Wang *et al.* investigated Ni–Co/Al_2_O_3_ catalysts with varying Ni/Co ratios in a water-cooled DBD reactor operated at 35 °C and 0.1 MPa, using a CO_2_ : H_2_ ratio of 1 : 3, a total flow rate of 32 mL min^−1^, a frequency of 9.2 kHz, and 500 mg catalyst packed in a 50 mm discharge zone with a 1 mm discharge gap.^47^ The 7Ni3Co composition delivered the best performance, achieving 24% CO_2_ conversion, 46% methanol selectivity, and a methanol STY of 4.7 mmol g_cat_^−1^ h^−1^. *In situ* FTIR and DFT calculations suggested that the Ni–Co interfacial sites promote asymmetric CO_2_ adsorption and facilitate progressive hydrogenation toward methanol through COOH* intermediates. The bimetallic interface lowered the calculated barrier of the rate-limiting step to 0.63 eV and suppressed RWGS-derived CO desorption by favouring further hydrogenation.

Beyond conventional metal oxide supports such as alumina, Ni–Co synergy has also been explored using metal–organic frameworks as supports. Hosseini *et al.* compared Co/ZIF-67 and Ni–Co/ZIF-67 catalysts in a parallel-plate DBD plasma reactor operated at ambient temperature and pressure using a CO_2_ : H_2_ feed ratio of 1 : 3. The reactor consisted of two parallel stainless-steel electrodes (5 cm diameter) covered by 1 mm quartz dielectric barriers and separated by an optimised 2.5 mm discharge gap. Approximately 0.5 g of catalyst was packed between the electrodes and excited using a 50 Hz AC power supply operated at a constant discharge power of 2 W and an input voltage of 10 kV.^62^ As shown in Fig. 15, Co/ZIF-67 achieved 69.6% CO_2_ conversion and 84.1% methanol selectivity. Introducing Ni further improved performance. The bimetallic Ni–Co/ZIF-67 catalyst reached 90.3% conversion and 98.1% methanol selectivity, and the energy efficiency increased from 2.7 to 4.8 mmol kJ^−1^. Both catalysts retained stable performance over six reuse cycles. The improved activity of the Ni–Co system was attributed to synergistic interactions between Ni and Co that enhanced adsorption and activation of CO_2_ and H_2_ and shifted selectivity toward methanol formation. As can be seen from Tables 2 and 3, this is the highest CO_2_ conversion, methanol selectivity and STY reported to date for plasma-catalytic CO_2_ hydrogenation to methanol. However, direct comparison with other studies remains difficult due to differences in reactor configuration and operating conditions.

**Fig. 15:**
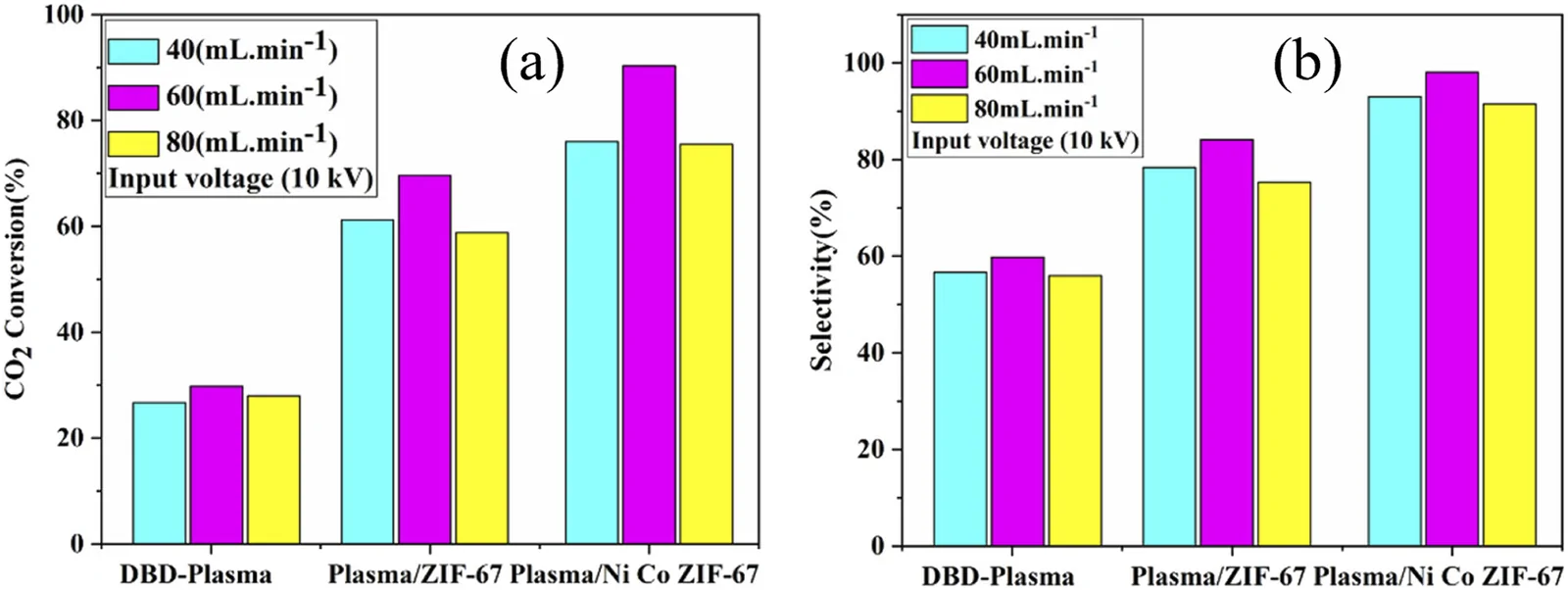
Performance of Ni–Co/ZIF-67 catalysts prepared by Hosseini *et al.* in plasma-assisted CO_2_ hydrogenation (CO_2_ : H_2_ = 1 : 3). (a) CO_2_ conversion, and (b) selectivity in different flow rates. Adapted with permission from ref. 62. Copyright 2018 American Chemical Society.

**Table 3:** Summary of the bimetallic and multicomponent catalysts and their performance for plasma-assisted CO_2_ hydrogenation to methanol under optimised operating conditions reported in the literature

Catalyst	Temp. (°C)	SEI a (kJ L^−1^)	CO_2_ : H_2_	WHSV b (mL g^−1^ h^−1^)	*X*_CO_2__ c (%)	S_MeOH_^d^ (%)	MeOH STY e (mmol g_cat_^−1^ h^−1^)	MeOH EE f (mmol kWh^−1^)	Ref.
Ni–Co/ZIF-67	Ambient	2	1 : 3	7200	90.3	98.1	71.1	17 280	62
Pd–Fe_2_O_3_/γ-Al_2_O_3_	Ambient	11.8	1 : 4	60 000	16.3	26.5	23.1	—	64
Pt/film/In_2_O_3_	30	45	1 : 3	4800	37.0	62.6	11.1	—	63
CuO/Fe_2_O_3_/QW	200	1.2	1 : 3	20 000	16.7	32.7	9.3	—	61
Cu–Zn/ZSM-5	20	21	1 : 3	7740	15	37.4	5.1	—	54
Ni–Co/Al_2_O_3_	35	15	1 : 3	3840	24.0	46.0	4.7	—	47
Pt/In_2_O_3_	30	45	1 : 3	4800	24.9	36.5	4.5	—	63
Cu/ZnO/Al_2_O_3_	30	45	1 : 3	4800	25.6	35.1	4.4	—	63
Cu–Ni/γ-Al_2_O_3_–CeO_2_	150	21	1 : 4.5 g	19 200	27.5	34.7	2.1	103.1	51
CuO–MgO/Beta	30	20	1 : 3	2400	8.5	72.0	2.0	201.1	41
NiO/Fe_2_O_3_/QW	200	1.2	1 : 3	20 000	1.7	33.3	1.2	—	61
Cu–Zn/MCF	25	25.1	1 : 3^h^	20 000	17.5	0.9	0.3	136.4	56
Cu–Co_3_O_4_	29	13.2	1 : 3^i^	15 000	15.3	0.4	0.1	1.5	42
Cu/ZnO/Al_2_O_3_	190	37.5	1 : 3	—	22.4	20.9	—	—	53

a Specific energy input.

b Weight hourly space velocity.

c CO_2_ conversion.

d Methanol selectivity.

e Methanol space-time yield.

f Energy efficiency.

g CO_2_ : H_2_ : Ar = 1 : 3 : 4.

h CO_2_ : H_2_ : Ar = 1 : 3 : 1.

i CO_2_ : H_2_ : Ar = 1 : 3 : 1.

Noble metals have received comparatively less attention than Cu- or Co-based catalysts in plasma-catalytic methanol synthesis, but notable performance enhancements have been reported when Pt is integrated with oxide supports and interfacial modifiers. Men *et al.* developed a Pt/film/In_2_O_3_ catalyst in which highly dispersed Pt nanoparticles were embedded within a peptide-derived conductive film deposited on In_2_O_3_.^63^ In a DBD reactor operated at atmospheric pressure and 30 °C using a CO_2_ : H_2_ ratio of 1 : 3 and a total gas flow rate of 40 mL min^−1^, the catalyst achieved 37.0% CO_2_ conversion and 62.6% methanol selectivity, outperforming conventionally prepared Pt/In_2_O_3_ and commercial Cu/ZnO/Al_2_O_3_ benchmarks. The improved performance was attributed primarily to the smaller Pt particle size and higher Pt dispersion achieved through room-temperature plasma-assisted preparation, which suppressed nanoparticle aggregation and increased the Pt–support interfacial area. In addition, the peptide film enhanced CO_2_ adsorption through conductive and proton-containing surface functionalities. Additionally, FTIR studies indicated enhanced formation of carbonate, bicarbonate, and methoxy surface species associated with methanol synthesis.

Tang *et al.* also reported a plasma-assisted Pd–Fe_2_O_3_/Al_2_O_3_ catalyst prepared using fluidised-bed plasma-assisted atomic layer deposition.^64^ The catalyst was evaluated in a packed-bed DBD reactor operated at atmospheric pressure using a CO_2_ : H_2_ ratio of 1 : 4, a discharge power of 19.6 W, a gas hourly space velocity (GHSV) of 10 000 h^−1^, and an AC excitation frequency of 27.7 kHz. The reactor consisted of a quartz tube (9 mm inner diameter) with a coaxial stainless-steel high-voltage electrode and was loaded with 100 mg catalyst. The catalyst showed higher CO_2_ conversion and methanol selectivity than the corresponding monometallic Pd or Fe_2_O_3_ systems under DBD plasma conditions. XPS and H_2_-TPR analyses indicated strong electronic interactions between Pd and Fe species. The authors proposed that charge transfer between the two components enhanced the adsorption and activation of CO_2_ and hydrogen-containing intermediates. The Pd–Fe_2_O_3_ catalyst achieved a CO_2_ conversion of 16.3% with methanol selectivity of 26.5% under plasma conditions, which was attributed to synergistic interactions between Pd, Fe_2_O_3_, and the plasma environment. Representative bimetallic and multicomponent catalysts investigated for plasma-catalytic CO_2_ hydrogenation to methanol are summarised in Table 3.

## 5. Operating parameters affecting performance

### 5.1. Reactor configuration and discharge characteristics

Reactor configuration plays an important role in determining the performance of plasma-catalytic CO_2_ hydrogenation to methanol because it directly influences the discharge behaviour, plasma-catalyst contact, and local reaction environment. Among the various NTP systems, DBD reactors are the most widely employed due to their simple design, atmospheric-pressure operation, and compatibility with catalyst integration.^26,51^ Packed-bed plasma-catalysis generally provides higher methanol selectivity than plasma-alone systems because the catalyst is positioned directly within the discharge region, enabling strong interactions between plasma-generated species and catalyst surfaces. Under these conditions, plasma species such as vibrationally excited CO_2_, H radicals, CO, and ions can participate directly in surface hydrogenation reactions before undergoing gas-phase recombination.^50,51^

Within packed-bed DBD systems, electrode geometry and reactor dimensions influence the discharge environment. Annular coaxial reactors and planar reactors differ in electrode curvature, discharge-gap geometry and available plasma-catalyst contact area. The inner and outer radii of an annular bed, or the plate spacing and electrode area in a planar configuration, together with the discharge length, determine the available discharge volume. Changing these dimensions at fixed gas flow and discharge power also changes the nominal residence time and power density. Consequently, equal applied voltages or total powers do not necessarily produce equivalent plasma environments. Modelling studies have demonstrated that discharge-gap dimensions strongly influence electric fields, electron properties and discharge behaviour.^65^

The physical properties of catalyst supports and packing materials are also important. Dielectric polarisation can enhance local electric fields near particle contacts, influencing electrical breakdown and the distribution of microdischarges. Particle dimensions, contact geometry and interparticle voids further determine the available discharge channels. In addition, catalyst packing can modify the local electric field and alter the discharge pattern from volume discharge to surface discharge, thereby affecting plasma chemistry and the participation of reactive species in surface reactions.^51^ Experimental single-pellet studies have shown that dielectric constant and applied voltage affect discharge modes and the extent of plasma formation.^66^ However, a higher dielectric constant does not necessarily improve overall performance. Modelling predicts that stronger local fields can coexist with reduced plasma coverage and lower average electron density, depending on the reactor geometry.^65^ Support effects should therefore be interpreted through both their catalytic properties and their influence on the discharge. Reactor geometry also affects the discharge mode. Wang *et al.* showed that a water-cooled DBD reactor with strong filamentary discharges produced higher methanol yields than reactors with weaker or more diffuse discharges.^26^ Water cooling maintained near-ambient temperatures (∼30 °C), which suppressed further decomposition of produced methanol and favoured oxygenate formation, as higher operating temperatures were found to shift product distribution toward CO rather than methanol.

Intraparticle porosity introduces another level of complexity. Pore dimensions influence reactant transport and access to internal active sites, but also affect local electric fields and the possibility of discharge formation within pores. Modelling studies have predicted microdischarges inside micrometre-sized pores and, under particular pulsed-discharge conditions, nanometre-sized pores. Their formation and spatial distribution depend on pore dimensions, applied voltage and the discharge conditions.^44,67^ These calculations concern specific gases and idealised geometries. A high specific surface area therefore does not by itself demonstrate uniform plasma access to internal catalytic sites, and an optimum pore size for plasma-catalytic methanol synthesis remains to be established.

Fluidised-bed DBD reactors require additional consideration because particle motion, bed expansion and changing solids distributions can modify gas–solid contact and the discharge environment. Pan *et al.*, for example, used Ar flow to fluidise Co_3_O_4_/Al_2_O_3_–PTFE particles within a coaxial DBD reactor during methanol synthesis.^48^ In a separate study of CO_2_ dissociation, Maina *et al.* demonstrated that fluidised-particle loading affected discharge intensity and conversion, with excessive loading causing discharge damping.^68^ These findings highlight the importance of considering solids loading and particle distribution. Comparisons between packed-bed and fluidised-bed methanol systems should therefore account for differences in hydrodynamics, gas dilution and plasma exposure alongside catalyst composition.

These results highlight the coupled influence of discharge characteristics and thermal management on methanol production, and the need to evaluate reactor configuration together with catalyst properties and operating conditions.

### 5.2. Feed composition and gas atmosphere

The feed gas composition strongly influences discharge parameters, reactive species generation, and product distribution during plasma-catalytic CO_2_ hydrogenation. Variations in the CO_2_ : H_2_ molar ratio directly affect reduced electric field intensity, breakdown voltage, and mean electron energy, with higher CO_2_ fractions increasing these parameters and promoting vibrational excitation and electron-induced CO_2_ dissociation.^69^ Most studies employ a stoichiometric CO_2_ : H_2_ ratio of 1 : 3 for methanol synthesis (as reflected in Tables 2 and 3), as higher H_2_ content enhances electron density and generally improves methanol selectivity and CO_2_ conversion efficiency under plasma conditions.^69,70^

Excessively high CO_2_ content in the feed gas can be counterproductive under plasma conditions. As CO_2_ content increases, the concentration of O and OH radicals generated through plasma-induced CO_2_ dissociation also increases. Microkinetic modelling on Cu(111) predicted when the partial pressure of O species exceeds approximately half that of H radicals in the plasma, the catalyst surface becomes progressively poisoned by strongly adsorbed O*, since the primary consumption pathway for O*, reaction with surface H*, becomes too slow to compensate for the rapid O* accumulation. This O* poisoning severely suppresses the CH_3_OH turnover frequency and shifts product distribution toward CO rather than methanol. Therefore, an optimal feed composition should maintain a sufficiently high H radical concentration relative to O species to keep the catalyst surface free of O* poisoning while sustaining adequate CO_2_ activation.^50^

The surrounding gas atmosphere and the presence of additional species can also affect reaction performance. Gas additives such as Ar and N_2_ can also influence discharge behaviour and product distribution during plasma-assisted CO_2_ hydrogenation. The nature of the noble gas can significantly influence product distribution during plasma-assisted CO_2_ hydrogenation. Masumbuko *et al.* investigated Ar and N_2_ addition to a non-catalytic DBD system containing H_2_, CO_2_ and CO. The addition of Ar decreased CO_2_ conversion by approximately 8% relative to the additive-free mixture and reduced methanol selectivity from 64.9% to 43%, while CH_4_ selectivity increased from 35% to 39.5%. In contrast, N_2_ resulted in substantially higher methanol production, with the authors attributing the difference partly to the different third-body efficiencies of N_2_ and Ar in plasma-phase reactions. Although both gases produced a more homogeneous discharge, Ar showed no tangible reduction in plasma power demand and appeared to favour reaction pathways associated with methanation rather than methanol formation.^71^

Ar is also frequently incorporated into the feed of plasma-catalytic methanol studies without its individual contribution being systematically evaluated. For example, Chen *et al.* employed 20 vol% Ar as part of the CO_2_ : H_2_ feed during plasma-catalytic methanol synthesis over Cu/MCF catalysts,^56^ while Fang *et al.* used a feed containing 20 vol% CO_2_, 60 vol% H_2_ and balance Ar in a DBD reactor over Cu–Co_3_O_4_ catalysts.^42^ Pan *et al.* employed substantial Ar dilution to facilitate catalyst fluidisation in a fluidised-bed DBD reactor.^48^ However, because these studies did not independently vary the Ar concentration, its specific contribution to CO_2_ conversion and methanol selectivity cannot be separated from the effects of catalyst composition and other operating parameters.

The presence of H_2_O in the reaction environment can influence both reaction performance and catalyst stability. Surface water accumulation can suppress methanol selectivity by blocking CO_2_ adsorption and hydrogenation pathways. As discussed in Section 4.1.2, physically mixing Co_3_O_4_/Al_2_O_3_ with hydrophobic PTFE improved methanol yield by promoting water desorption and limiting the occupation of active sites by H_2_O.^48^ Water can also affect catalyst stability. Evidence from conventional thermal methanol synthesis shows that water can accelerate Al_2_O_3_ sintering in Cu/ZnO/Al_2_O_3_ catalysts, causing support degradation, changes in Cu, ZnO and Cu/ZnO phases, and growth of Cu particles.^72,73^ Water-assisted formation of copper carbonate species has also been reported as a deactivation mechanism in some Cu-based methanol synthesis catalysts.^74^

However, water can also promote methanol formation in suitable plasma-catalytic systems. Pan *et al.* reported that H_2_O addition in combination with oxygen-vacancy-rich CoO catalysts enhanced methanol selectivity through the generation of additional H and OH radicals under plasma conditions, which promoted methanol formation *via* both CO stepwise hydrogenation and direct CH_3_ and OH coupling pathways.^49^ Cui *et al.* also observed increased methanol selectivity following H_2_O addition over Cu/γ-Al_2_O_3_. Their calculations attributed this promotional effect to competitive adsorption that weakened methanol binding and facilitated its desorption.^39^ Complementary evidence from thermal CO_2_ hydrogenation over Cu–ZnO–ZrO_2_ showed that water facilitated the hydrolysis of surface methoxy intermediates formed through the formate pathway. Enhanced water transport through macroporous catalyst structures also improved methanol selectivity in this system.^13^ Calculations for Pd–Cu alloy surfaces further suggested that small amounts of water formed *in situ* through RWGS and methanol synthesis could lower barriers for O–H bond formation and increase methanol turnover frequency.^75^

These promotional effects must be balanced against inhibition. Increasing water content can reduce CO_2_ conversion through competitive adsorption, changes in discharge characteristics and competing electron–impact processes involving H_2_O. Consequently, the effect of water depends on its concentration, the catalyst and the plasma operating conditions. Water addition should therefore be evaluated using methanol yield and energy efficiency alongside selectivity, since improved selectivity can be offset by lower CO_2_ conversion.

### 5.3. Energy input and residence time

Discharge power, applied voltage, and residence time strongly affect electron energy, plasma density, and reactant activation. Increasing discharge power generally enhances CO_2_ conversion as higher-energy electrons promote vibrational excitation and electron-impact dissociation of CO_2_.^69^ However, higher power does not necessarily improve methanol selectivity, since elevated specific energy input can favour CO formation through the RWGS reaction or promote over-hydrogenation toward methane.^26,49^ An optimal discharge power therefore exists that balances CO_2_ activation with selective surface hydrogenation toward methanol.

Gas flow rate and residence time also influence product distribution by controlling the duration of plasma exposure and contact between reactive species and catalyst surfaces. Lower flow rates and longer residence times generally improve CO_2_ conversion by increasing the probability of electron-molecule collisions.^69^ However, the effect on methanol selectivity is less straightforward, as methanol yield has been reported to be relatively insensitive to flow rate changes in some systems, and excessively long residence times may promote secondary gas-phase reactions.^26^ Since discharge characteristics, reactor geometry, and catalyst packing differ significantly between studies, direct comparison of operational parameters remains challenging. Consequently, systematic optimisation of plasma conditions together with catalyst design remains essential for improving methanol productivity and selectivity.^26,51,69^

## 6. Challenges, opportunities and future outlook

Plasma-catalytic CO_2_ hydrogenation to methanol has shown significant progress in recent years, particularly in mechanistic understanding and catalyst development. NTP enables CO_2_ activation under relatively mild conditions and offers opportunities for electrified and decentralised methanol production. However, the field remains at an early stage, and several scientific and technological challenges still limit practical implementation.

From the catalysis point of view, one of the major challenges in plasma-catalytic CO_2_ hydrogenation is the incomplete understanding of plasma-catalyst interactions under reaction conditions. Although catalyst restructuring also occurs in thermocatalytic systems, plasma catalysis can be even more dynamic and introduce additional non-equilibrium effects that further complicate catalyst behaviour under reaction conditions. These plasma-induced changes can alter active sites, influence adsorption behaviour, and modify reaction pathways and product selectivity. A further unresolved issue in this context is identifying the optimal balance between plasma-driven activation and surface-mediated hydrogenation. Under low plasma intensity, insufficient CO_2_ activation limits overall conversion, whereas at higher discharge power, enhanced CO formation *via* RWGS can suppress methanol selectivity. Defining this optimal plasma-catalyst operating window will be essential for improving process performance. Consequently, the relationship between catalyst structure, plasma properties, and methanol formation is not fully understood. This in turn limits the rational design of plasma-specific catalysts. That is why many studies rely partly on empirical optimisation or adaptation of conventional thermocatalytic materials. Advanced *in situ* and *operando* characterisation techniques, including DRIFTS and X-ray absorption spectroscopy, plasma diagnostics and theoretical modelling, can play a critical role in identifying the dynamic structural and chemical changes occurring during plasma operation. Closer integration of these approaches will be essential for establishing predictive plasma-catalyst-structure–activity relationships and developing rational catalyst design principles for plasma-assisted methanol synthesis.

Another important challenge is the relatively low energy efficiency reported for many plasma-catalytic systems. NTP enables operation near ambient conditions, but a substantial fraction of the electrical energy can still be lost through non-productive excitation, gas heating, or undesired side reactions. Direct comparisons are complicated by differences in operating conditions and system boundaries. Nevertheless, Chen *et al.* reported an energy efficiency of 5.32% for plasma-catalytic CO_2_ hydrogenation under single-pass operation and compared this value with a literature-reported energy efficiency of 35.7% for conventional thermocatalytic methanol synthesis. However, direct comparison should be made with caution because energy-efficiency calculations may depend on the system boundaries, energy inputs considered, and evaluation methodology adopted in each study.^51^ Although this single comparison cannot be taken as broadly representative, it illustrates that improving energy efficiency remains one of the most critical challenges for plasma-catalytic systems to approach industrial relevance.

A related challenge that is only beginning to receive attention is the techno-economic feasibility of plasma-catalytic CO_2_ hydrogenation to methanol at scale. Recent techno-economic analysis (TEA) studies suggest that current plasma-catalytic systems remain economically uncompetitive relative to conventional thermocatalytic methanol synthesis, primarily because of high electricity consumption and plasma reactor capital costs.^76,77^ Nevertheless, these studies also highlight the environmental advantages of plasma-driven systems when coupled with renewable electricity, including net-negative carbon emissions demonstrated under defined renewable energy supply assumptions. However, existing analyses remain heavily dependent on laboratory-scale reactor performance data and practical considerations such as CO_2_ capture, feed impurities, and catalyst deactivation behaviour are typically excluded from the defined system boundaries. Comprehensive process-level TEA and life cycle assessment (LCA) under industrially realistic conditions therefore remain largely limited. Such analyses will be essential for identifying the energy efficiency, CO_2_ conversion, and methanol selectivity targets required for industrial implementation of plasma-catalytic methanol synthesis. In addition, the cost and source of hydrogen supply will strongly influence the overall feasibility and sustainability of plasma-driven methanol synthesis.

Meaningful comparison between plasma-catalytic studies also remains difficult because of variations in reactor configuration, power measurement methods, residence times, catalyst packing, and reporting metrics. Standardised protocols for evaluating CO_2_ conversion, methanol selectivity, energy efficiency, and plasma power consumption would greatly improve benchmarking across studies and accelerate rational development of plasma-catalytic systems.

Reactor scale-up remains another important challenge. DBD reactors are relatively simple and may be scalable through modular or numbering-up approaches, which can help preserve the discharge characteristics of small-scale systems. However, maintaining consistent plasma behaviour, stable operation, and reproducible plasma-catalyst interactions across multiple parallel reactor units remains difficult, particularly in packed-bed DBD configurations where local electric fields and microdischarge formation are highly sensitive to reactor geometry and catalyst packing. In addition, most studies still rely on high-purity CO_2_ and H_2_ under controlled laboratory conditions. Practical deployment will require compatibility with realistic CO_2_ streams derived from flue gases, biogas upgrading, fermentation, or direct air capture. Such feeds may contain impurities including O_2_, H_2_O, SO_*x*_, NO_*x*_, hydrocarbons, amines, and particulates, which could influence discharge behaviour, methanol selectivity, and catalyst stability. The tolerance of plasma-catalytic systems to such impurities remains largely unexplored.

Despite these challenges, plasma-catalysis also presents several opportunities. Improved understanding of these plasma-catalyst interactions could also open new opportunities for catalyst design. Interface engineering, oxygen vacancy control, bifunctional active sites, and emerging strategies such as tuning surface hydrophobicity all appear promising for directing plasma-assisted hydrogenation pathways toward methanol. From the studies reviewed here, bimetallic and multicomponent catalysts often achieve higher methanol productivity and selectivity than monometallic systems. However, these catalysts are typically more complex to synthesise and may increase material and processing costs.

An important opportunity for plasma-catalytic systems lies in their compatibility with intermittent renewable electricity sources. Owing to their low thermal inertia, plasma reactors can be started and stopped rapidly and can operate under dynamic conditions, making them promising candidates for flexible, decentralised methanol production powered by renewable electricity.

Looking ahead, future research should focus not only on maximising catalytic performance, but also on developing simpler, scalable, and cost-effective catalyst systems with high methanol productivity under plasma conditions. Further progress will also depend on closer integration of *operando* characterisation, plasma diagnostics, and theoretical modelling to establish predictive plasma-catalyst structure–activity relationships and reduce reliance on empirical catalyst development.

Future progress in this field may also be accelerated through the application of artificial intelligence and machine learning. Plasma catalysis involves a large number of strongly interdependent variables, including catalyst composition and architecture, plasma power, reactor geometry, gas composition, and catalyst packing properties. Data-driven approaches could help identify relationships between catalyst descriptors and reaction performance, optimise operating conditions, and accelerate catalyst discovery. Combining machine learning with *operando* characterisation, plasma diagnostics, density functional theory calculations, and kinetic modelling may reduce the current reliance on empirical approaches. Combining artificial intelligence with *operando* characterisation, plasma diagnostics, density functional theory calculations, and kinetic modelling may reduce the current reliance on empirical approaches. Advancing plasma energy efficiency, deepening mechanistic understanding of plasma-catalyst interactions, and designing catalysts with higher methanol selectivity will determine whether plasma-catalytic methanol synthesis can move beyond the laboratory.

## Author contributions

Maryam Khatibi: conceptualization, funding acquisition, writing – original draft, writing – review & editing. Jie Gu: writing – review & editing. Paul T. Williams: writing – review & editing. Amin Osatiashtiani: conceptualization, project administration, writing – original draft, writing – review & editing. During the preparation of this work the authors used Copilot and ChatGPT in order to improve the language. After using this tool/service, the authors reviewed and edited the content as needed and take full responsibility for the content of the publication.

## Conflicts of interest

The authors declare that they have no known competing financial interests or personal relationships that could have appeared to influence the work reported in this paper.

## Data Availability

No primary research results, software or code have been included and no new data were generated or analysed as part of this review.
